# Inequalities in disease burden and care quality of chronic obstructive pulmonary disease, 1990–2021: Findings from the Global Burden of Disease Study 2021

**DOI:** 10.7189/jogh.14.04213

**Published:** 2024-09-27

**Authors:** Lingzi Yao, Jin Cao, Siqing Cheng, Shiyi Shan, Denan Jiang, Zeyu Luo, Shuting Li, Leying Hou, Xue Li, Peige Song

**Affiliations:** Center for Clinical Big Data and Statistics of the Second Affiliated Hospital Zhejiang University School of Medicine, School of Public Health Zhejiang University School of Medicine, Hangzhou, China

## Abstract

**Background:**

Chronic obstructive pulmonary disease (COPD) is one of the primary causes of significant morbidity and mortality worldwide. This study aimed to explore the cross-country inequalities by age, sex, and region in COPD’s burden and care quality from 1990 to 2021.

**Methods:**

We obtained data from the Global Burden of Disease 2021. Using age-standardised disability-adjusted life years rate (ASDR) per 100 000 population and quality of care index (QCI), we quantified the COPD burden and care quality, respectively. Applying the principal component analysis method, we calculated QCI scores, ranging from 0 to 100, where higher values indicate better care quality. We quantified temporal trends from 1990 to 2021 for ASDR and QCI by estimated annual percentage change (EAPC). Finally, we assessed the absolute and relative disparities in ASDR and QCI across countries using the slope index of inequality (SII) and concentration index.

**Results:**

Between 1990 and 2021, there was a notable decline in ASDR of COPD globally (1990 = 1492.64; 2021 = 940.66; EAPC = −1.71), accompanied by an increase in QCI (1990 = 58.42; 2021 = 73.86; EAPC = 0.89). Regions with middle sociodemographic index (SDI) consistently demonstrated the highest ASDR and the lowest QCI in 1990 (ASDR = 2332.91; QCI = 31.70), whereas by 2021, low-middle SDI regions exhibited similar trends (ASDR = 1707.90; QCI = 57.50). In 2021, the highest ASDR was among individuals aged 95 years and above (16251.22), while the lowest QCI was among people aged 70–74 years (72.18). Papua New Guinea recorded the highest ASDR and the lowest QCI in 2021 (ASDR = 3004.36; QCI = 19.18). Compared to 1990, where the SII for ASDR was −612.44 and for QCI was 21.78, with concentration indices of −0.14 for ASDR and 0.11 for QCI, the absolute values of both SII and concentration index were smaller in 2021, with ASDR’s SII at −555.90, QCI’s at 16.72, ASDR’s concentration index at −0.13, and QCI’s at 0.04.

**Conclusions:**

The global burden of COPD decreases and care quality increases over time, with notable variations across ages, sexes and SDI regions. Countries with lower SDI had disproportionately higher burden and poorer care quality for COPD.

Chronic obstructive pulmonary disease (COPD) is characterised by persistent respiratory symptoms and airflow limitation due to airway and/or alveolar abnormalities, primarily caused by exposure to toxic particles or gases [1]. Ranking as the third leading cause of mortality worldwide, COPD represents a significant global health challenge, with approximately 3.23 million deaths attributed to it in 2019 [2]. Beyond mortality, COPD also significantly contributes to morbidity [3], and is the 7th leading cause of disabilities globally [2]. Such fatal and non-fatal burden of COPD exert a profound impact on individual well-being and public health systems [2,3]. The economic burdens are striking as well. In 2010, the global cost of COPD amounted to 2.1 trillion US dollars (USD), with projections suggesting a doubling of this expense by 2030 [4]. Notably, the burden of COPD is not uniformly distributed across populations but shows marked variations by age, sex, and region [5]. Emerging research has shown that regions with lower sociodemographic development levels experience a greater impact from COPD [6], underscoring the need to investigate the intersection between COPD epidemiology and inequality.

The World Health Organization (WHO) defines quality of care as the degree to which health services increase the likelihood of desired health outcomes in individuals and populations, achieved through efficient, effective, and safe delivery [7]. Optimal care quality is essential to mitigate the burden of COPD. Despite the availability of cost-effective interventions, COPD management is often deprioritised relative to other non-communicable diseases such as cardiovascular diseases and cancer [8]. Previous studies have indicated that socioeconomic disparities contribute to the variations in health outcomes related to COPD [9], with the global concentration of wealth further entrenching these health inequalities. One reason behind this might be disparities in access to and quality of health care services [10]. For instance, over the past thirty years, age-standardised incidence, prevalence, mortality, and disability-adjusted life years (DALYs) rates for COPD have steadily increased in sub-Saharan Africa, a partial reflection of inadequate health services and fragile health systems [11]. Conversely, China has experienced a decline in the age-standardised DALYs rate (ASDR) for COPD from 1990 to 2019, potentially due to improvements in health care accessibility through the widespread implementation of basic medical insurance [12]. Hence, to better quantify the burden of disease and quality of care, and more specifically meet the needs of individuals, it is essential to identify the differences in health indicators across countries/territories of varying socioeconomic development.

Our study aimed to simultaneously analyse the burden and quality of care for COPD on a global scale from 1990 to 2021 to identify countries or regions with a high disease burden yet sub-optimal care quality, serving as priority targets for global health interventions. Additionally, by investigating the extent of cross-country inequalities, we sought to not only quantify the absolute and relative disparities in COPD burden and quality of care using the slope index of inequality (SII) and concentration index but also uncovered the disparities among countries with varying socio-economic development levels.

## METHODS

### Data source

The Global Burden of Disease (GBD) 2021 study presents comprehensive epidemiological estimates for 371 diseases and injuries by age and sex across 204 countries and territories, offering annual data from 1990 to 2021 on a global, regional, and national scale [13]. Detailed descriptions of the methodologies have been reported, and fatal and non-fatal estimates have been published (https://vizhub.healthdata.org/gbd-compare/ and https://ghdx.healthdata.org/gbd-results-tool). For this study, we extracted annual COPD-related incidence, prevalence, deaths, DALYs, years of life lost (YLLs), and years lived with disability (YLDs) by age, sex, region and country/territory for the period 1990–2021 from the Global Health Data Exchange (GHDx) query tool (https://vizhub.healthdata.org/gbd-results/). Sociodemographic index (SDI) is a summary measure scaled from 0 to 1, representing the social and economic conditions for health outcomes in each location. It encompasses a spectrum of societal variables, including the fertility rate among the population aged below 25, the educational attainment of individuals aged 15 and above, and per capita income and closely associated with health-related outcomes [14]. Based on their SDIs, all available 204 countries and territories were categorised into five sociodemographic development quintiles (low, low-middle, middle, high-middle, and high) [14].

### Case definition

The definition of COPD adheres to the Global Initiative for Chronic Obstructive Lung Disease (GOLD) guidelines: a value of <0.7 for forced expiratory volume in one second/forced vital capacity (one second of forceful exhalation/total forced expiration) with spirometry, after bronchodilation [15]. International Classification of Diseases 10th Revision codes associated with COPD include J41-J44. The corresponding International Classification of Diseases 9th Revision codes are 491–492 and 496. Estimation of COPD burden was based on 203 original sources, including literature, hospital claims data, proportion data of GOLD, and Burden of Obstructive Lung Disease (BOLD) study data [14].

### Quality of care index

In our evaluation of the quality of care for COPD in various countries, we utilised the quality of care index (QCI), a composite index that had been shown in previous studies to effectively assess care quality by incorporating multiple outcome indicators [16]. This index integrates four secondary metrics – mortality to incidence ratio (MIR), DALYs to prevalence ratio, prevalence to incidence ratio, and YLLs to YLDs ratio – derived from six primary COPD outcome measures. These four ratios could be formulated as:

MIR = Age-standardised death rate/Age-standardised incidence rate (1)

DALYs to prevalence ratio = Age-standardised DALYs rate/Age standardised prevalence rate (2)

Prevalence to incidence ratio = Age-standardised prevalence rate/Age-standardised incidence rate (3)

YLLs to YLDs ratio = Age-standardised YLLs rate/Age-standardised YLDs rate (4)

A principal component analysis (PCA) was conducted to condense these metrics into a single composite QCI score, scaled from 0 to 100, with higher scores indicating better quality of care[17]. The age-standardised QCI could be formulated as:

Age-standardised QCI = (PCAscore–minPCAscore)/(maxPCAscore–minPCAscore) (5)

### Cross-country health inequality of burden and care quality

The analysis of cross-country health inequality, including the inequality of disease burden and care quality, delineated the disparities and variations in disease burden and care quality indices among numerous nations based on their national averages, which could be informed by the data derived from GBD [18]. We employed two standardised metrics, SII and concentration index, to quantitatively assess the distributional inequality of ASDR and QCI of COPD across countries [18], as mentioned in the statistical analysis section later on.

### Statistical analysis

#### Descriptive analysis and trend analysis of burden and care quality

Age-standardised metrics were applied to enable valid comparisons of COPD burden and care quality across different demographic profiles and temporal dimensions. Age categories commenced from 15–19 years, with subsequent intervals of five years, extending to those aged 95 and above. We used ASDR of COPD for 100 000 population and QCI to represent the burden and care quality of COPD respectively, by sex, year, and SDI quintiles.

To delineate the progression of COPD’s ASDR, we calculated estimated annual percent change (EAPC), a prevalent metric for gauging the trajectory of age-standardised rates across a specified period [19–21]. The parameter β in equation (6) was derived from fitting the regression equation: ln(ASR) = α + βx + ε, with x representing the year. The 95% confidence interval (CI) for the EAPC was ascertained by incorporating the lower and upper bounds of the 95% CI for β from equation (6) [22]. The ASDR was interpreted as having risen if the EAPC’s lower boundary of the 95% CI exceeded 0, and similarly, it was interpreted as having fallen if this boundary was less than 0. When the 95% CI encompassed zero, the ASDR was deemed to have remained unchanged [23].

EAPC = 100 × (e^β^ − 1) (6)

#### Cross-country inequality analysis

We computed SII by a regression of national ASDR in the entire population on a relative position scale associated with the SDI, which was defined as the midpoint of the cumulative SDI-ranked population range [24]. To account for heteroskedasticity, a weighted regression model was employed. A positive SII indicated higher ASDR or QCI with increasing SDI, while a negative SII signified the opposite. The extent of health inequality was indicated by the SII value, with larger values denoting greater disparities between high and low SDI countries.

Concentration index was calculated by numerically integrating the area under the Lorenz curve, constructed based on the cumulative fraction of ASDR and QCI and the cumulative relative distribution of the population ranked by SDI [25]. Depending on the position of the curve in relation to the line of equality (45-degree line), it indicated that the health indicator was higher in lower-SDI countries/territories when the curve exceeded this line, while higher-SDI countries/territories had a higher ASDR or QCI when the curve was situated below it. Concentration index varied from −1 to 1, with negative value signifying a greater concentration of health indicator within low SDI countries/territories, and positive value denoting a concentration in high SDI countries/territories.

All analyses and visualisations were conducted in *R*, version 4.3.1 (2023 Frederiksberg, Denmark). All statistical tests were two-sided, and *P*-values less than 0.05 were considered statistically significant.

## RESULTS

### The burden of COPD from 1990 to 2021

**Figure 1:**
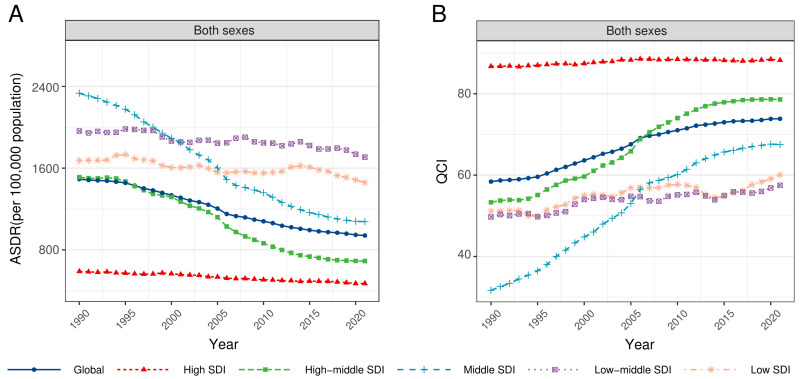
Temporal trend of ASDR (**Panel A**) and QCI (**Panel B**) of COPD from 1990 to 2021. ASDR – age-standardised DALYs rate (per 100 000 population), COPD – chronic obstructive pulmonary disease, DALYs – disability-adjusted life years, QCI – quality of care index, SDI – sociodemographic index.

**Figure 2:**
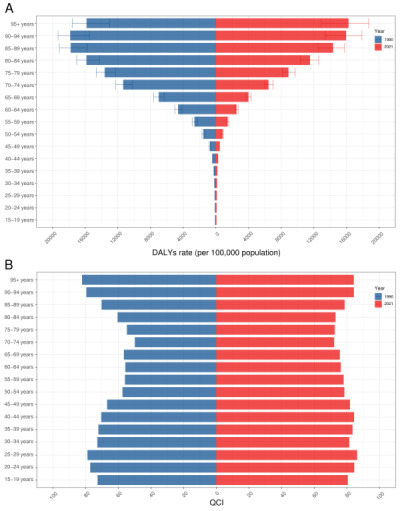
Age distribution of global DALYs rate (**Panel A**) and QCI (**Panel B**) in 1990 and 2021. COPD – chronic obstructive pulmonary disease, DALYs – disability-adjusted life years, QCI – quality of care index.

**Figure 3:**
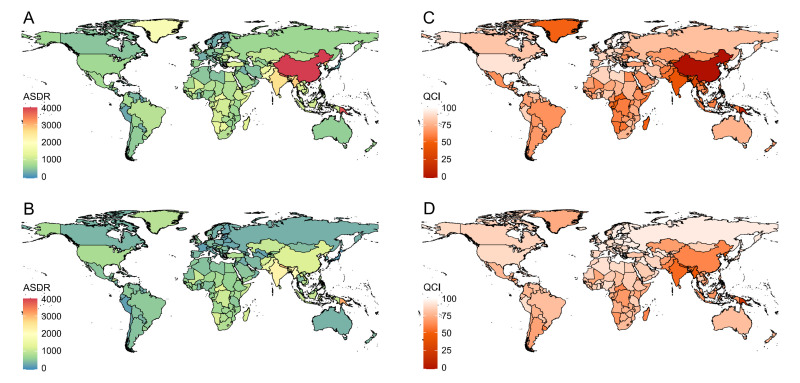
Global distribution of ASDR and QCI of COPD in 1990 and 2021. **Panel A**. ASDR in 1990. **Panel B.** ASDR in 2021. **Panel C.** QCI in 1990. **Panel D.** QCI in 2021. ASDR – age-standardised DALYs rate (per 100 000 population), COPD – chronic obstructive pulmonary disease, DALYs – disability-adjusted life years, QCI – quality of care index.

Globally, ASDR of COPD was reduced from 1990 (1492.64 per 100 000 population, 95% uncertainty interval (UI) = 1342.46, 1609.30) to 2021 (940.66 per 100 000 population, 95% UI = 871.48, 1014.59), with the EAPC of −1.71 (95% CI = −1.79, −1.63) (**Table 1**, **Figure 1**). For people who aged 15–44, DALYs rate remained consistently low, while those who were older than 80 took the overwhelming majority in DALYs rate of COPD. The highest DALYs rate was observed among individuals aged 90−94 years in 1990, reaching 17829.24 (95% UI = 15 464.57, 19 323.76), and by 2021, this rate was highest among individuals aged 95 and above, reaching 16 251.22 (95% UI = 12 905.46, 18 697.90). (**Figure 2**, Table S1 in the **Online Supplementary Document**). As for sex differences, ASDR of COPD was higher in males than females in both 1990 (male = 1937.66 per 100 000 population, 95% UI = 1737.83, 2127.01; female = 1155.29 per 100 000 population, 95% UI = 934.32, 1299.04) and 2021 (male = 1177.27 per 100 000 population, 95% UI = 1047.92, 1288.57; female = 750.60 per 100 000 population, 95% UI = 661.98, 848.24) (**Table 1**). Across SDI regions, middle SDI regions had the highest ASDR in 1990 (2332.91 per 100 000 population, 95% UI = 2063.49, 2546.31) while low-middle SDI regions had the highest ASDR in 2021(1707.90 per 100 000 population, 95% UI = 1558.88, 1865.11). In contrast, high SDI regions persistently had the lowest ASDR from 1990 (589.80 per 100 000 population, 95% UI = 557.84, 616.39) to 2021 (471.22 per 100 000 population, 95% UI = 437.45, 498.84) (**Table 1**, **Figure 1**). The top five countries/territories with the highest ASDR were China, Papua New Guinea, Nepal, Democratic People’s Republic of Korea, and Myanmar in 1990, and shifted to Papua New Guinea, Nepal, India, Democratic People’s Republic of Korea and Myanmar in 2021 (**Figure 3**, Table S2 in the **Online Supplementary Document**). Specific ASDR data were presented in Tables S1–2, Figures S1–2 in the **Online Supplementary Document**.

**Table 1:** ASDR and QCI of COPD in 1990 and 2021 and the EAPC from 1990 to 2021

Variables	ASDR (95% UI)			QCI		
	**1990**	**2021**	**EAPC**	**1990**	**2021**	**EAPC**
	**NO. (95%UI)**	**NO. (95%UI)**	**(95%CI)**			**(95%CI)**
Global						
Both sexes	1492.64 (1342.46, 1609.30)	940.66 (871.48, 1014.59)	−1.71 (−1.79, −1.63)	58.42	73.86	0.89 (0.82, 0.96)
*Female*	1155.29 (934.32, 1299.04)	750.60 (661.98, 848.24)	−1.63 (−1.73, −1.54)	61.52	74.69	0.76 (0.70, 0.82)
*Male*	1937.66 (1737.83, 2127.01)	1177.27 (1047.92, 1288.57)	−1.82 (−1.89, −1.74)	58.34	74.10	0.89 (0.82, 0.96)
SDI region						
*High SDI*	589.80 (557.84, 616.39)	471.22 (437.45, 498.84)	−0.76 (−0.80, −0.71)	86.75	88.26	0.07 (0.05, 0.08)
*High-middle SDI*	1511.32 (1365.74, 1635.67)	691.14 (621.83, 772.74)	−3.06 (−3.25, −2.87)	53.33	78.61	1.54 (1.43, 1.65)
*Middle SDI*	2332.91 (2063.49, 2546.31)	1076.67 (963.62, 1201.24)	−2.83 (−2.94, −2.72)	31.70	67.53	2.71 (2.49, 2.93)
*Low-middle SDI*	1963.19 (1602.24, 2252.75)	1707.90 (1558.88, 1865.11)	−0.38 (−0.44, −0.32)	49.72	57.50	0.43 (0.36, 0.49)
*Low SDI*	1673.81 (1373.37, 1936.99)	1457.94 (1318.76, 1617.05)	−0.38 (−0.46, −0.30)	51.18	60.06	0.46 (0.36, 0.56)

ASDR – age-standardised DALYs rate (per 100 000 population), CI – confidence interval, COPD – chronic obstructive pulmonary disease, DALYs –disability-adjusted life years, EAPC – estimated annual percentage change, QCI – quality of care, UI – uncertainty interval, SDI – sociodemographic index

### The care quality of COPD from 1990 to 2021

From 1990 to 2021, QCI of COPD increased globally (1990 = 58.42; 2021 = 73.86), with the EAPC of 0.89 (95% CI = 0.82, 0.96) (**Table 1**, **Figure 1**). In both 1990 and 2021, people aged 70–74 years (1990 = 49.87; 2021 = 72.18) had the lowest QCI (**Figure 2**, Table S1 in the **Online Supplementary Document**). Females had a marginally higher QCI than males in both 1990 (female = 61.52; male = 58.34) and 2021 (female = 74.69; male = 74.10) (**Table 1**). High SDI regions consistently had the highest QCI from 1990 (86.75) to 2021 (88.26), while other SDI regions exhibited upward trends in care quality of COPD from 1990 to 2021, with the middle SDI regions experiencing the most substantial increase (**Table 1**, **Figure 1**). Middle SDI region had the lowest care quality of COPD in 1990 (31.70), while in 2021, low-middle SDI region had the lowest (57.50). The top five countries/territories with the lowest QCI were China, Papua New Guinea, Nepal, Nauru and Myanmar in 1990, and became Papua New Guinea, Nepal, Nauru, Vanuatu and Myanmar in 2021 (**Figure 3**, Table S3 in the **Online Supplementary Document**). Specific QCI data were presented in Tables S1, S3, Figures S1, S3 in the **Online Supplementary Document**.

### Cross-country inequality of COPD burden

**Figure 4:**
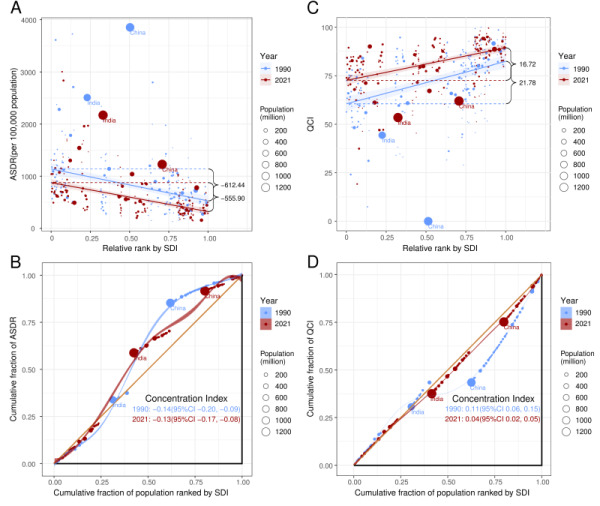
Global distribution of ASDR and QCI of COPD in 1990 and 2021. **Panel A.** SII for DALYs. **Panel B.** concentration index for DALYs. **Panel C.** SII for QCI. **Panel D**. Concentration index for QCI.SII – slope index of inequality. ASDR – age-standardised DALYs rate (per 100 000 population), COPD – chronic obstructive pulmonary disease, DALYs – disability-adjusted life years, QCI – quality of care index, SDI – sociodemographic index.

Significant absolute SDI-related inequalities in ASDR of COPD were observed, showing a disproportionately higher burden shouldered by countries with lower SDI. As shown by SII, the disparity in ASDR between the highest and lowest SDI country/territory in 2021 (−555.90, 95% CI = −670.85, −440.95) was smaller than in 1990 (−612.44, 95% CI = −793.80, −431.08) (**Figure 4**, Table S4 in the **Online Supplementary Document**). Moreover, the concentration index, which indicated relative inequalities, was −0.13 (95% CI = −0.17, −0.08) in 2021, which was smaller than the 1990 value (−0.14, 95% CI = −0.20, −0.09) (Table S4, Figure S4 in the **Online Supplementary Document**).

### Cross-country inequality of COPD care quality

Significant absolute SDI-related inequalities in QCI of COPD were found. Countries/territories with lower SDI levels had a lower QCI level. Furthermore, it was noteworthy that that the inequality of QCI was different between 1990 and 2021. Specifically, SII demonstrated that the disparity between high SDI and low SDI countries/territories was smaller in 2021 in comparison with 1990. For instance, as presented by SII analysis, QCI disparity between the highest SDI and lowest-SDI countries was 21.78 (95% CI = 15.89, 27.68) in 1990 and 16.72 (95% CI = 12.69, 20.75) in 2021, which suggested the disparity between QCI levels in countries/territories with different social development levels was narrower in 2021. As for relative inequalities, Lorenz curves of QCI for COPD intersected with the equality line both in 1990 and 2021 while most of the curves lay below the quality line, indicating a slight privilege of health care quality in high SDI countries/territories. Concentration index was 0.11 (95% CI = 0.06, 0.15) in 1990 and 0.04 (95% CI = 0.02, 0.05) in 2021, signifying that the disparities in global health care quality were less pronounced in 2021 (**Figure 4**, Table S5 in the **Online Supplementary Document**).

## DISCUSSION

To the best of our knowledge, this is the first study that seeks to answer the question of the global trends in ASDR and care quality for COPD as well as their cross-country inequality based on a novel secondary analysis of the GBD 2021 data set. From 1990 to 2021, the ASDR of COPD decreased globally, with variations across ages, sexes, SDI regions and countries. QCI scores showed a steady global increase in quality of care for COPD from 1990 to 2021, with people aged 70–74 having lower quality of care. Health inequality analysis revealed that DALYs of COPD were predominantly concentrated within economically impoverished nations. Countries/territories with higher SDI had a better quality of care.

From 1990 to 2021, ASDR of COPD showed a downward trend, signifying significant progress was achieved in reducing the burden of COPD. DALYs rate of COPD kept increasing with age, which could be attributed to the factors below. The older individuals are more prone to accumulated exposure to risk factors such as smoking and environmental pollutants over their lifetime, which significantly increases the likelihood of developing COPD [26]. Besides, older individuals are at a higher risk of developing comorbidities, such as cardiovascular diseases, which can exacerbate COPD and contribute to a more significant overall disease burden [27]. Moreover, the natural decline in immune function with age makes older adults more susceptible to respiratory infections that can trigger or worsen COPD exacerbations [28].

Our research indicated that regions with higher SDI had superior health care quality, which might be associated with the low DALYs of COPD observed in this study. Despite notable improvements observed in QCI for regions with low and low-middle SDI, a significant disparity remains when compared to areas with higher SDI. Studies affirmed that increased health care expenditures were correlated with enhanced health care quality [29]. Notably, nations characterised by low SDI demonstrated limited resources, low education and awareness, insufficient social support and low health care expenditures, explaining the discernible QCI differentiation from those nations with high SDI [30,31].

The most significant QCI was observed among individuals aged 25–29 and those above 90. Given that QCI encompassed incidence as a variable, this observation aligned with the documented lower incidence of COPD within younger generations [32]. As for elderly individuals, it was imperative to acknowledge that QCI scores exhibit suboptimal reliability within the elder age group, especially for those beyond 90 years [33]. This could be attributed to the fact that fewer years have accounted for deaths within this age group. The diminished loss of years consequently attenuates YLLs and DALYs in proportion to the elevated age-specific incidence and prevalence of COPD within this demographic.

Disease burden inequality analysis indicated that DALYs of COPD were primarily concentrated in lower SDI regions. Tobacco use is a significant risk factor for COPD [34], while the number of smokers in the high-income areas had a substantial relative decrease since 1990 [35]. Meanwhile, developing countries tended to have greater biomass consumption, which was also a significant cause for COPD [36]. Besides, inadequate health care resources and infrastructure in low SDI regions might result in COPD patients facing challenges in diagnosis and treatment, further aggravating the burden of COPD [37]. Furthermore, as a significant risk factor for COPD, air pollution levels are higher in low SDI regions, further contributing to the concentration of COPD disease burden in these areas [38].

As for QCI inequality, both SII and concentration index were positive, showing that higher QCI scores tended to be distributed in regions with higher SDI levels, accounting for adequate medical resources and health expenditure [30,31]. Both the SII and concentration index implied that the disparities in QCI levels among countries with different levels of social development decreased from 1990 to 2021. As measured before, QCI score had been increasing in middle SDI, low-middle SDI, and low SDI regions, while the disparities in health care quality between rich and poor areas were diminishing, reducing the SII and concentration index [39].

With the assistance of GBD 2021 and other advanced computational tools, our study’s strengths lie in its robust research design, substantial and diversified sample size, and the utilisation of advanced statistical methods. Our research systematically delineated the impact of COPD disease burden and quality of care across different age groups, SDI regions, and countries/territories. Moreover, our study, utilising the SII and concentration index methods, underscored the disparities in DALYs and quality of care across different nations, providing a direct opportunity for resource allocation assessment.

Our study revealed significant disparities in the burden and quality of care for COPD across different socioeconomic regions, necessitating targeted health policy interventions and strategic resource allocation. Specifically, regions with lower SDI experienced higher COPD burdens and lower quality of care, as previously discussed, regions with low SDI had higher rates of smoking, biomass consumption, and air pollution [35–38], and were characterised by limited medical resources, lower levels of health education, insufficient social support, and lower health care expenditure [30,31,39]. Therefore, increased investment in health infrastructure and training was required to improve the accessibility of health care in these regions, and to enhance public health campaigns to reduce exposure to risk factors such as smoking and air pollution [37]. Concurrently, strengthening the primary health care system for early diagnosis and management of COPD could improve patient outcomes [40]. China had significantly enhanced the quality of care for chronic diseases, including COPD, by expanding the coverage of basic medical insurance, strengthening the capacity of primary health care services, and implementing national chronic disease prevention and control programmes [41]. This comprehensive health system reform had improved the efficiency and accessibility of medical services, providing a valuable example for the global community. Moreover, the research findings emphasised the need to establish global health partnerships to provide financial and technical assistance to strengthen medical resources and infrastructure in low SDI regions, thereby alleviating health inequality and improving the management of COPD. Additionally, to formulate effective policies and evaluate interventions, improving data collection and monitoring systems was crucial. These measures would help more accurately track health inequality related to COPD and support locally tailored interventions, thereby reducing the burden of COPD within a broader socioeconomic context [42]. These recommendations, based on our results, aimed to guide policymakers in reducing disparities and achieving more equitable health outcomes globally.

Our study was also subjected to several inherent limitations. First, using data derived from samples may only partially represent part of the studied country or territory [14]. Due to the inadequacies in certain underdeveloped regions’ health care systems, data collection might be susceptible to inherent biases that could influence the accuracy of the estimates. Second, variations in diagnostic criteria for airway obstruction (e.g. expiratory volume in one second/forced vital capacity <0.70 or the lower limit of normal) might trigger variability in COPD diagnosis rates [43]. Additionally, the reliance on age-standardised metrics, while enabling cross-country comparisons, might not fully capture the nuances and complexities of each nation’s health care system and disease epidemiology, including incomplete data, differences in age structures, challenges in considering individual differences, and an inability to fully reflect the complexity of diseases [44]. Furthermore, QCI index might be significantly influenced by the substantial heterogeneity in health care infrastructure, resource allocation, and sociocultural factors across nations. A single, overarching index might not adequately capture the multidimensional aspects of health care quality, necessitating more granular and context-specific evaluation methods such as cluster analysis or latent class analysis that could avoid the potentially obscuring effect of principal component analysis on these nuances [45]. Moreover, our analysis did not thoroughly examine or control for potential confounders that could affect the relationship between socioeconomic development and COPD burden, such as health care infrastructure, cultural differences in smoking habits, and regional variations in air pollution, which may also introduce potential bias in cross-country comparisons of socioeconomic estimates [46]. These factors could significantly impact the observed disparities and should be considered in future research to enhance the validity of the conclusions regarding socioeconomic disparities. Nonetheless, this study has potential implications for public health interventions targeting COPD, offering guidance for developing more precise health policies and allocating health care resources.

## CONCLUSIONS

In summary, though the ASDR of COPD has been decreasing across countries, COPD is still a significant public health problem globally, with several inequalities in care and outcomes. Disparities in the global COPD burden and health care resources necessitate collaborative efforts among governments worldwide to address these challenges. We hope the findings and insights from this study will guide further reforms in COPD health care strategies, resource allocation, and policy development.

## Additional material


Online Supplementary Document
