# Supplementary material for: Inequalities in disease burden and care quality of chronic obstructive pulmonary disease, 1990–2021: Findings from the Global Burden of Disease Study 2021
Source: J Glob Health. 2024 Sep 27;14:04213. doi: 10.7189/jogh.14.04213 (PMC11428470; doi:10.7189/jogh.14.04213)
Supplement: Online Supplementary Document [file jogh-14-04213-s001.pdf]

ONLINE SUPPLEMENTARY DOCUMENT

Title: Inequalities in disease burden and care quality of chronic obstructive pulmonary disease, 1990-2021: findings from the Global Burden of Disease Study 2021

Authors: Lingzi Yao, Jin Cao, Siqing Cheng, Shiyi Shan, Denan Jiang, Zeyu Luo, Shuting Li, Leying Hou, Xue Li, Peige Song

Table S1. The age-specific DALYs rate and QCI of COPD worldwide and across different SDI regions

Notes: DALYs – disability-adjusted life years; QCI – quality of care index; COPD – chronic obstructive pulmonary disease; SDI – sociodemographic index.

Table S2. ASDR of COPD at the national level in 1990 and 2021 and the EAPC from 1990 to 2021

Notes: ASDR – age-standardised DALYs rate (per 100 000 population); DALYs – disability-adjusted life years; COPD – chronic obstructive pulmonary disease; EAPC – estimated annual percentage change; UI – uncertainty interval; CI – confidence interval.

Table S3. QCI of COPD at the national level in 1990 and 2021 and the EAPC from 1990 to 2021

Notes: QCI – quality of care index; EAPC – estimated annual percentage change; COPD – chronic obstructive pulmonary disease; CI – confidence interval.

Table S4. The cross-country inequalities in ASDR of COPD in 1990 and 2021, by sex

Notes: ASDR – age-standardised DALYs rate (per 100 000 population); DALYs – disability-adjusted life years; COPD – chronic obstructive pulmonary disease; CI – confidence interval.

Table S5. The cross-country inequalities in QCI of COPD, by sex

Notes: QCI – quality of care index; COPD – chronic obstructive pulmonary disease; CI – confidence interval.

Fig S1. Year trend of ASDR (A) and QCI (B) of COPD in global and different SDI regions

Notes: ASDR – age-standardised DALYs rate (per 100 000 population); DALYs – disability-adjusted life years; QCI – quality of care index; COPD – chronic obstructive pulmonary disease; SDI – sociodemographic index.

Fig S2. The age-specific DALYs rates of COPD in global and different SDI regions in 1990 (A) and 2021 (B)

Notes: DALYs – disability-adjusted life years; COPD – chronic obstructive pulmonary disease; SDI – sociodemographic index.

Fig S3. The age-specific QCI of COPD in global and different SDI regions in 1990 (A) and 2021 (B)

Notes: QCI – quality of care index; COPD – chronic obstructive pulmonary disease; SDI – sociodemographic index.

Fig S4. The cross-country inequalities in ASDR of COPD in 1990 and 2021, by sex

Notes: SII – slope index of inequality; COPD – chronic obstructive pulmonary disease; ASDR – age-standardised DALYs rate (per 100 000 population); DALYs – disability-adjusted life years. Panel A-C: SII (A. Both; B. Female; C. Male); Panel D-F: concentration index (D. Both; E. Female; F. Male).

Fig S5. The cross-country inequalities in QCI of COPD in 1990 and 2021, by sex

Notes: QCI – quality of care index; SII – slope index of inequality; COPD – chronic obstructive pulmonary disease; SDI, sociodemographic index. Panel A-C: SII (A. Both; B. Female; C. Male); Panel D-F: concentration index (D. Both; E. Female; F. Male).

Table S1. The age-specific DALYs rate and QCI of COPD worldwide and across different SDI regions

SDI regions	1990			2021		
	Age	DALYs rate	QCI	Age	DALYs rate	QCI
Global	15-19	60.62	72.71	15-19	42.35	80.52
	20-24	82.20	77.17	20-24	59.11	84.48
	25-29	106.20	78.89	25-29	79.15	86.23
	30-34	152.97	72.85	30-34	107.58	81.48
	35-39	233.56	72.18	35-39	147.04	83.45
	40-44	399.72	70.46	40-44	240.19	84.36
	45-49	733.01	66.86	45-49	434.17	81.84
	50-54	1535.97	57.42	50-54	827.86	78.44
	55-59	2625.16	55.92	55-59	1444.03	77.97
	60-64	4623.55	55.71	60-64	2534.89	76.24
	65-69	6999.09	56.60	65-69	3996.35	75.64
	70-74	11350.29	49.87	70-74	6459.28	72.18
	75-79	13610.78	54.73	75-79	8897.92	72.57
	80-84	15844.82	60.45	80-84	11529.47	73.02
	85-89	17777.15	70.26	85-89	14354.64	78.61
	90-94	17829.24	79.55	90-94	15946.10	84.24
	95+	15828.96	82.20	95+	16251.22	84.20
High SDI	15-19	21.71	86.41	15-19	17.28	88.72
	20-24	29.48	91.40	20-24	24.06	92.38
	25-29	39.37	94.54	25-29	32.54	93.17
	30-34	55.34	89.55	30-34	46.39	91.52
	35-39	76.89	91.06	35-39	65.24	92.97
	40-44	123.70	94.34	40-44	106.65	95.43
	45-49	254.44	91.29	45-49	217.19	92.65
	50-54	519.21	87.93	50-54	452.92	89.56
	55-59	973.72	85.95	55-59	835.22	88.83
	60-64	1805.50	83.32	60-64	1441.45	86.48
	65-69	2881.51	82.89	65-69	2149.84	87.67
	70-74	4410.43	84.73	70-74	3115.21	89.46
	75-79	5609.33	87.21	75-79	4290.52	90.56
	80-84	6466.50	85.96	80-84	5119.63	88.49
	85-89	7371.53	88.93	85-89	6593.86	90.19
	90-94	8027.31	92.24	90-94	8326.77	92.12
	95+	8431.92	92.02	95+	9650.82	90.56
High-middle SDI	15-19	53.58	73.46	15-19	29.33	83.58
	20-24	74.78	77.68	20-24	43.25	87.26
	25-29	92.36	80.89	25-29	55.93	90.97
	30-34	137.79	73.78	30-34	77.85	85.25
	35-39	217.35	72.30	35-39	108.10	86.90
	40-44	387.18	68.24	40-44	167.94	88.58

Table S1. The age-specific DALYs rate and QCI of COPD worldwide and across different SDI regions

SDI regions	1990			2021		
	Age	DALYs rate	QCI	Age	DALYs rate	QCI
	45-49	674.96	66.12	45-49	278.70	88.59
	50-54	1343.40	59.46	50-54	513.47	86.56
	55-59	2363.61	58.01	55-59	865.18	87.76
	60-64	3940.30	59.53	60-64	1480.92	85.88
	65-69	6286.32	57.97	65-69	2440.60	84.13
	70-74	11423.64	45.59	70-74	4201.95	80.21
	75-79	14140.78	48.58	75-79	6710.06	76.98
	80-84	18389.63	51.06	80-84	9392.46	75.76
	85-89	22959.04	59.51	85-89	14468.84	77.70
	90-94	25935.60	68.50	90-94	17507.97	82.36
	95+	22158.43	73.88	95+	20393.72	80.58
Middle SDI	15-19	73.67	67.17	15-19	38.57	81.21
	20-24	100.52	71.49	20-24	57.95	83.95
	25-29	127.84	72.85	25-29	76.89	86.08
	30-34	194.05	65.41	30-34	106.12	81.37
	35-39	310.18	62.93	35-39	144.78	83.54
	40-44	550.61	55.90	40-44	230.79	83.83
	45-49	926.62	55.23	45-49	403.40	82.40
	50-54	2056.83	40.61	50-54	769.84	79.26
	55-59	3466.83	40.13	55-59	1322.03	80.14
	60-64	6341.72	39.33	60-64	2414.73	77.04
	65-69	10112.51	38.30	65-69	4059.70	74.70
	70-74	17600.64	22.41	70-74	7111.16	68.34
	75-79	23440.50	22.88	75-79	10441.12	66.90
	80-84	28266.37	32.52	80-84	15197.88	66.07
	85-89	34239.28	44.68	85-89	21169.60	70.44
	90-94	35900.50	58.42	90-94	25040.98	76.75
	95+	29250.57	66.18	95+	24538.05	77.92
Low-middle SDI	15-19	62.57	74.80	15-19	48.35	79.88
	20-24	91.20	77.80	20-24	71.13	83.11
	25-29	131.84	76.69	25-29	100.53	83.97
	30-34	188.28	70.40	30-34	143.21	77.60
	35-39	276.74	70.31	35-39	204.19	78.35
	40-44	484.93	70.22	40-44	364.78	78.04
	45-49	972.17	62.89	45-49	710.22	73.21
	50-54	2106.03	50.06	50-54	1475.86	65.38
	55-59	3645.34	45.23	55-59	2772.45	58.89
	60-64	6909.43	41.75	60-64	4828.75	59.49
	65-69	10078.89	44.74	65-69	7766.78	58.51
	70-74	14082.26	42.86	70-74	12797.10	51.50

Table S1. The age-specific DALYs rate and QCI of COPD worldwide and across different SDI regions

SDI regions	1990			2021		
	Age	DALYs rate	QCI	Age	DALYs rate	QCI
	75-79	17323.20	46.60	75-79	16116.19	55.41
	80-84	19951.32	55.26	80-84	21256.35	57.73
	85-89	20168.18	70.08	85-89	22808.97	70.87
	90-94	21173.64	79.02	90-94	25391.28	78.81
	95+	23955.82	77.66	95+	26691.97	78.51
Low SDI	15-19	71.60	72.42	15-19	58.73	76.74
	20-24	85.83	80.15	20-24	74.25	82.89
	25-29	122.92	79.79	25-29	105.24	83.22
	30-34	174.58	73.43	30-34	148.33	77.18
	35-39	250.13	74.02	35-39	198.19	79.68
	40-44	425.89	73.31	40-44	317.26	80.97
	45-49	906.15	62.04	45-49	645.90	74.16
	50-54	1832.16	52.31	50-54	1227.01	70.19
	55-59	3155.27	48.07	55-59	2273.23	65.27
	60-64	5782.32	45.20	60-64	4193.01	62.80
	65-69	8680.58	43.64	65-69	6826.37	60.05
	70-74	11926.46	40.71	70-74	11257.91	52.29
	75-79	14193.75	44.95	75-79	13153.86	56.92
	80-84	16912.63	52.68	80-84	17703.03	56.50
	85-89	17175.87	67.10	85-89	19385.55	67.83
	90-94	17609.76	76.24	90-94	19863.48	76.75
	95+	21545.59	72.44	95+	21587.45	74.75

Notes: DALYs – disability-adjusted life years; QCI – quality of care index; COPD – chronic obstructive pulmonary disease; SDI – sociodemographic index.

Table S2. ASDR of COPD at the national level in 1990 and 2021 and the EAPC from 1990 to 2021

Countries /Territories	1990 NO. (95%UI)	2021 NO. (95%UI)	EAPC NO. (95%CI)	Countries /Territories	1990 ASDR NO. (95%UI)	2021 NO. (95%UI)	EAPC NO. (95%CI)
Papua New Guinea	3724.74 (2788.16, 4811.98)	3004.36 (2404.29, 3732.82)	-0.69 (-0.73 to -0.64)	Libya	473.66 (369.66, 595.71)	504.59 (410.08, 628.23)	0.53 (0.38 to 0.67)
Nepal	3611.26 (2493.00, 4552.90)	2836.01 (2275.31, 3485.04)	-0.65 (-0.86 to -0.44)	Cuba	356.98 (335.91, 378.45)	501.11 (445.80, 556.48)	1.07 (0.89 to 1.26)
India	2508.84 (1975.51, 2965.31)	2171.16 (1953.69, 2422.39)	-0.36 (-0.46 to -0.26)	Norway	316.34 (296.03, 338.19)	497.3 (456.34, 532.24)	1.46 (1.08 to 1.84)
Democratic People's Republic of Korea	2799.28 (2073.73, 3681.84)	1968.20 (1500.48, 2612.25)	-1.13 (-1.24 to -1.03)	Burkina Faso	568.38 (478.52, 665.91)	491.7 (416.02, 586.83)	-0.40 (-0.46 to -0.33)
Myanmar	2710.62 (2042.45, 3435.53)	1958.92 (1604.09, 2354.00)	-1.32 (-1.43 to -1.21)	Nigeria	590.98 (473.38, 708.27)	490.54 (424.15, 569.06)	-0.59 (-0.64 to -0.55)
Vanuatu	2338.24 (1539.15, 3050.72)	1709.11 (1132.62, 2218.10)	-1.20 (-1.27 to -1.12)	Algeria	530.38 (447.94, 630.53)	488.58 (415.38, 573.16)	-0.09 (-0.16 to -0.02)
Kiribati	2197.67 (1410.58, 3256.15)	1684.31 (1265.06, 2595.52)	-0.97 (-1.03 to -0.90)	Argentina	568.44 (534.79, 605.11)	487.71 (454.18, 519.70)	-0.43 (-0.67 to -0.18)
Bhutan	2374.53 (1683.60, 3107.79)	1678.89 (1317.32, 2166.00)	-1.30 (-1.41 to -1.18)	Saint Lucia	487.08 (458.95, 523.15)	487.49 (404.87, 574.54)	-0.42 (-0.64 to -0.20)
Lesotho	1355.16 (1050.28, 1752.63)	1616.31 (1182.32, 2071.61)	1.10 (0.77 to 1.43)	Mauritania	757.41 (599.92, 899.98)	482.86 (395.79, 574.92)	-1.35 (-1.66 to -1.03)
Republic of Nauru	2233.05 (1739.46, 3176.44)	1614.56 (1176.72, 2426.49)	-1.18 (-1.35 to -1.02)	Lebanon	700.15 (509.11, 888.11)	477.84 (416.89, 545.89)	-0.94 (-1.08 to -0.81)
Pakistan	1783.98 (1421.94, 2048.46)	1541.67 (1287.52, 1855.86)	-0.68 (-0.90 to -0.46)	Belgium	691.93 (649.94, 728.78)	477.58 (437.24, 512.88)	-1.35 (-1.46 to -1.25)
Marshall Islands	2150.42 (1637.24, 2544.26)	1467.49 (1126.82, 1862.39)	-1.14 (-1.21 to -1.06)	Serbia	679.95 (593.41, 787.76)	475.15 (406.91, 548.67)	-1.28 (-1.38 to -1.19)
Federated States of Micronesia	2353.05 (1717.15, 2906.41)	1396.63 (1121.89, 1761.24)	-1.80 (-1.91 to -1.69)	Plurinational State of Bolivia	667.98 (546.14, 789.81)	470.77 (376.96, 590.85)	-1.01 (-1.04 to -0.97)
Samoa	1995.61 (1503.27, 2539.75)	1396.39 (1123.60, 1732.57)	-1.19 (-1.28 to -1.10)	Bolivarian Republic of Venezuela	401.81 (373.82, 426.23)	462.77 (370.70, 574.17)	0.23 (-0.06 to 0.52)
Solomon Islands	1791.80 (1431.40, 2207.36)	1368.16 (1142.77, 1653.18)	-0.89 (-0.97 to -0.82)	Thailand	1179.10 (1010.81, 1352.99)	461.07 (381.24, 559.88)	-3.70 (-3.95 to -3.44)
Republic of Palau	1639.14 (1348.08, 1990.14)	1338.13 (1120.27, 1589.76)	-0.56 (-0.62 to -0.50)	Tunisia	463.75 (400.76, 538.26)	460.89 (370.65, 585.52)	-0.10 (-0.15 to -0.04)
Central African Republic	1514.36 (1022.39, 1953.54)	1302.89 (863.86, 1797.34)	-0.55 (-0.59 to -0.50)	Romania	1072.32 (1010.94, 1131.17)	459.19 (415.10, 503.41)	-2.70 (-2.99 to -2.42)
Bangladesh	2281.06 (1801.47, 2722.49)	1301.68 (1050.53, 1612.22)	-1.91 (-2.13 to -1.69)	Cyprus	921.04 (690.89, 1093.42)	455.12 (393.60, 521.74)	-2.37 (-2.56 to -2.17)
Sao Tome and Principe	1359.56 (1157.63, 1583.01)	1269.67 (1005.65, 1563.85)	-0.18 (-0.33 to -0.04)	The former Yugoslav Republic of Macedonia	599.24 (533.68, 688.26)	454.42 (362.35, 594.99)	-1.08 (-1.22 to -0.95)
Kingdom of Eswatini	1513.96 (1073.79, 1893.29)	1264.80 (979.92, 1626.08)	-0.21 (-0.65 to 0.23)	Mongolia	849.27 (694.29, 1029.74)	453.68 (376.58, 532.25)	-2.71 (-2.94 to -2.49)
China	3852.57 (3349.97, 4279.01)	1227.66 (1048.45, 1442.54)	-4.19 (-4.37 to -4.00)	Ireland	975.14 (917.99, 1023.41)	451.69 (406.89, 492.35)	-2.62 (-2.87 to -2.38)
Lao People's Democratic Republic	1862.13 (1252.31, 2347.83)	1176.83 (892.84, 1502.07)	-1.71 (-1.80 to -1.63)	Belize	294.92 (271.92, 318.85)	444.57 (391.58, 501.54)	1.01 (0.48 to 1.54)
Namibia	1431.13 (1088.79, 1767.53)	1171.78 (951.35, 1457.16)	-0.77 (-0.97 to -0.57)	Germany	489.59 (455.52, 521.56)	442.86 (409.16, 475.53)	-0.11 (-0.34 to 0.12)
Afghanistan	1426.64 (960.32, 1838.69)	1155.17 (875.13, 1480.07)	-0.84 (-1.05 to -0.63)	New Zealand	671.68 (638.18, 703.55)	431.82 (393.22, 462.12)	-1.73 (-1.88 to -1.57)
Madagascar	1263.53 (1025.66, 1497.36)	1147.81 (892.85, 1466.97)	-0.47 (-0.54 to -0.40)	Islamic Republic of Iran	478.53 (405.07, 540.13)	424.08 (383.16, 462.24)	-0.17 (-0.27 to -0.08)

Tuvalu	2060.08 (1495.59, 2496.17)	1138.27 (890.67, 1437.52)	-2.01 (-2.10 to -1.92)	Czech Republic	484.17 (443.76, 533.14)	414.71 (371.23, 462.55)	0.65 (0.21 to 1.1)
Democratic Republic of the Congo	1125.72 (811.56, 1536.86)	1073.41 (720.28, 1615.37)	-0.17 (-0.21 to -0.13)	Ghana	410.50 (348.32, 481.99)	411.82 (344.86, 486.00)	0.40 (0.16 to 0.65)
Honduras	887.48 (746.75, 1076.22)	1049.64 (846.10, 1295.33)	0.77 (0.59 to 0.94)	Croatia	444.88 (412.36, 478.73)	410.22 (367.21, 456.28)	-0.11 (-0.20 to -0.02)
Timor-Leste	1262.75 (875.76, 1586.04)	1047.94 (814.39, 1288.81)	-0.58 (-0.70 to -0.47)	Spain	662.52 (622.96, 704.42)	410.16 (370.27, 444.09)	-1.68 (-1.81 to -1.54)
Mali	1174.28 (980.64, 1375.82)	1042.23 (837.23, 1324.98)	-0.17 (-0.29 to -0.06)	Guam	862.24 (786.16, 943.49)	404.98 (359.51, 450.33)	-2.25 (-2.48 to -2.02)
Indonesia	1140.65 (870.70, 1312.20)	1040.41 (874.72, 1216.00)	-0.25 (-0.34 to -0.16)	Bosnia and Herzegovina	686.75 (620.88, 752.25)	403.71 (338.66, 474.43)	-2.01 (-2.20 to -1.82)
Somalia	1313.57 (868.25, 1712.30)	1040.23 (683.92, 1374.61)	-0.78 (-0.84 to -0.72)	Luxembourg	555.20 (521.72, 590.12)	399.59 (360.41, 442.07)	-1.08 (-1.17 to -0.99)
Kazakhstan	1010.63 (908.23, 1113.48)	1023.13 (889.04, 1171.56)	-0.43 (-0.99 to 0.14)	Iceland	477.90 (440.40, 517.30)	397.50 (353.63, 437.69)	-0.57 (-0.74 to -0.4)
American Samoa	1544.45 (1371.26, 1739.36)	993.97 (860.80, 1150.40)	-1.60 (-1.70 to -1.50)	Palestine	588.43 (467.45, 712.02)	397.17 (353.23, 454.73)	-1.43 (-1.62 to -1.24)
Tonga	1330.02 (1114.81, 1535.57)	988.89 (814.24, 1161.40)	-0.91 (-1.02 to -0.81)	Bulgaria	672.25 (608.95, 740.13)	394.48 (344.83, 447.94)	-1.82 (-1.95 to -1.68)
Republic of Niue	1496.95 (1271.72, 1761.46)	985.74 (811.57, 1191.85)	-1.63 (-1.72 to -1.54)	Canada	511.66 (478.41, 544.39)	393.18 (360.94, 423.66)	-0.97 (-1.09 to -0.86)
Northern Mariana Islands	1350.94 (1137.98, 1621.71)	940.60 (840.40, 1055.79)	-1.17 (-1.23 to -1.10)	Andorra	654.03 (509.97, 838.39)	390.98 (303.12, 486.99)	-1.54 (-1.76 to -1.31)
Cambodia	1118.74 (869.93, 1325.37)	935.16 (771.66, 1124.75)	-0.63 (-0.67 to -0.60)	Mauritius	683.52 (645.06, 723.59)	388.62 (361.01, 420.10)	-1.96 (-2.15 to -1.77)
Tokelau	1385.25 (982.56, 1830.78)	921.21 (704.89, 1227.57)	-1.52 (-1.59 to -1.46)	Republic of Cabo Verde	802.09 (669.17, 926.73)	388.20 (317.27, 462.46)	-2.21 (-2.81 to -1.62)
Greenland	1724.40 (1446.21, 1936.51)	907.57 (779.86, 1067.90)	-2.02 (-2.13 to -1.91)	Paraguay	347.01 (304.21, 401.38)	385.23 (315.58, 470.01)	0.54 (0.43 to 0.65)
Guinea-Bissau	1216.20 (911.72, 1492.77)	887.63 (660.38, 1092.06)	-0.71 (-0.92 to -0.51)	Nicaragua	374.25 (336.46, 413.67)	381.70 (327.58, 437.44)	0.44 (0.16 to 0.73)
Burundi	1309.37 (961.52, 1595.74)	877.53 (646.02, 1091.66)	-1.71 (-1.91 to -1.51)	Austria	380.49 (357.14, 406.30)	381.09 (350.49, 411.09)	0.24 (0.10 to 0.38)
Sri Lanka	1428.46 (1248.27, 1590.90)	864.19 (631.53, 1103.11)	-1.4 (-1.61 to -1.19)	Oman	488.48 (388.97, 597.76)	380.91 (326.15, 458.22)	-0.48 (-0.65 to -0.31)
South Sudan	1032.44 (731.85, 1333.20)	863.67 (616.81, 1149.37)	-0.8 (-1.03 to -0.57)	Greece	363.40 (330.46, 398.37)	380.35 (340.27, 424.19)	0.61 (0.13 to 1.09)
Socialist Republic of Viet Nam	1054.72 (810.90, 1299.01)	863.32 (702.77, 1024.54)	-0.6 (-0.67 to -0.52)	Costa Rica	487.63 (451.54, 518.91)	378.84 (336.42, 418.37)	-1.21 (-1.63 to -0.79)
South Africa	886.20 (796.06, 1027.79)	853.41 (789.28, 935.98)	-0.33 (-0.65 to 0.00)	Azerbaijan	663.27 (573.56, 768.07)	374.49 (309.17, 479.57)	-2.29 (-2.56 to -2.02)
Turkey	1199.96 (985.75, 1388.47)	850.19 (727.99, 983.51)	-0.90 (-1.15 to -0.64)	Dominica	359.73 (319.34, 400.09)	373.21 (326.91, 438.90)	0.14 (0.10 to 0.18)
Eritrea	1202.90 (861.07, 1497.37)	844.47 (676.17, 1047.84)	-1.18 (-1.26 to -1.10)	Australia	598.16 (564.83, 629.03)	365.77 (332.29, 390.19)	-1.69 (-1.92 to -1.47)
Botswana	1412.78 (1065.18, 1757.20)	840.65 (700.98, 1049.95)	-1.78 (-1.91 to -1.65)	El Salvador	472.52 (419.31, 539.06)	361.12 (294.30, 437.05)	-0.93 (-1.07 to -0.79)
Congo	1293.88 (892.44, 1768.41)	825.18 (673.44, 1037.16)	-1.70 (-1.81 to -1.59)	Suriname	418.99 (379.12, 465.36)	355.30 (284.80, 434.64)	-0.40 (-0.58 to -0.21)
Philippines	961.37 (882.15, 1056.81)	807.05 (707.12, 923.78)	-0.45 (-0.53 to -0.38)	Puerto Rico	422.95 (399.00, 448.54)	354.55 (305.58, 406.86)	-1.29 (-1.61 to -0.97)
Rwanda	1430.48 (1063.03, 1702.37)	800.01 (611.66, 1028.73)	-2.74 (-3.08 to -2.41)	Russian Federation	677.94 (654.80, 700.48)	352.01 (328.27, 379.88)	-2.74 (-3.05 to -2.43)

Fiji	1253.86 (1066.77, 1462.49)	778.59 (613.56, 940.77)	-2.01 (-2.22 to -1.79)	Guatemala	512.58 (471.00, 552.44)	348.63 (307.48, 392.08)	-1.33 (-1.61 to -1.05)
United States of America	723.91 (683.54, 765.50)	777.93 (725.17, 819.94)	0.22 (0.06 to 0.38)	Saint Kitts and Nevis	297.98 (274.17, 321.80)	345.50 (297.19, 389.88)	0.51 (0.36 to 0.65)
Kenya	715.37 (471.36, 1054.73)	766.86 (514.09, 1250.53)	0.42 (0.27 to 0.56)	Portugal	573.56 (539.50, 611.60)	344.54 (312.27, 373.10)	-1.83 (-2.04 to -1.61)
Chad	803.11 (598.74, 972.20)	763.57 (583.64, 942.36)	-0.02 (-0.1 to 0.07)	Taiwan (Province of China)	577.34 (539.63, 617.83)	343.42 (311.47, 377.54)	-1.94 (-2.11 to -1.77)
Republic of the Gambia	839.39 (665.37, 1026.54)	744.46 (564.13, 962.59)	-0.26 (-0.48 to -0.03)	Albania	759.54 (662.90, 864.71)	342.39 (280.66, 430.36)	-2.55 (-2.88 to -2.23)
Hungary	776.75 (734.04, 826.31)	744.21 (658.45, 834.29)	0.17 (-0.19 to 0.53)	Armenia	918.66 (869.63, 967.90)	339.94 (305.43, 379.96)	-3.16 (-3.59 to -2.72)
Yemen	905.15 (560.94, 1202.82)	730.21 (567.09, 920.09)	-0.73 (-0.81 to -0.66)	Sweden	314.04 (287.47, 343.60)	339.36 (304.58, 374.57)	0.27 (0.02 to 0.53)
Haiti	819.75 (392.57, 1165.65)	728.06 (356.16, 1071.15)	-0.25 (-0.31 to -0.20)	Qatar	605.95 (516.58, 706.51)	336.21 (287.54, 395.65)	-2.19 (-2.61 to -1.77)
Zimbabwe	644.41 (540.40, 755.08)	721.09 (593.96, 856.23)	0.63 (0.38 to 0.89)	Guyana	249.26 (226.01, 272.73)	336.02 (271.46, 415.63)	1.00 (0.79 to 1.20)
Guinea	802.39 (581.56, 985.96)	713.48 (558.98, 891.50)	-0.07 (-0.22 to 0.07)	Jamaica	281.79 (259.39, 300.73)	335.42 (271.26, 413.45)	0.31 (-0.19 to 0.81)
Zambia	782.84 (607.90, 948.22)	705.51 (572.04, 851.09)	-0.59 (-0.73 to -0.46)	Poland	597.58 (571.96, 626.59)	327.79 (298.61, 358.48)	-1.75 (-1.87 to -1.63)
Togo	816.20 (657.06, 956.45)	705.07 (558.67, 907.89)	-0.28 (-0.42 to -0.14)	Iraq	341.87 (280.61, 408.72)	320.73 (262.89, 381.13)	-0.53 (-0.64 to -0.41)
Malawi	744.64 (606.95, 885.90)	704.60 (592.39, 839.10)	-0.41 (-0.58 to -0.23)	Republic of Moldova	1001.85 (941.95, 1066.00)	314.75 (283.83, 348.95)	-4.33 (-4.71 to -3.94)
Niger	876.25 (635.55, 1086.55)	702.13 (524.46, 871.29)	-0.41 (-0.62 to -0.20)	Panama	361.93 (336.03, 389.55)	313.24 (258.10, 363.23)	-0.86 (-1.13 to -0.60)
Maldives	1882.94 (1269.67, 2256.62)	696.35 (597.42, 814.59)	-3.66 (-3.8 to -3.51)	Jordan	511.14 (436.06, 598.70)	311.01 (266.39, 360.78)	-2.03 (-2.25 to -1.81)
Uganda	979.47 (704.19, 1264.74)	693.40 (506.33, 880.76)	-1.59 (-1.75 to -1.43)	Grenada	260.65 (237.21, 286.14)	310.32 (274.60, 343.63)	0.28 (-0.10 to 0.67)
Angola	1133.66 (856.24, 1425.55)	692.53 (547.40, 863.01)	-1.87 (-1.95 to -1.79)	Turkmenistan	833.48 (757.54, 907.96)	307.79 (254.46, 369.50)	-3.97 (-4.54 to -3.40)
Mozambique	656.87 (524.85, 788.92)	683.39 (549.98, 823.52)	0.40 (0.27 to 0.53)	Ecuador	454.07 (427.00, 478.81)	296.86 (256.18, 345.10)	-0.90 (-1.11 to -0.68)
Denmark	798.05 (757.87, 841.24)	682.66 (625.85, 729.99)	-0.84 (-1.07 to -0.62)	Georgia	253.78 (227.99, 281.96)	295.55 (261.43, 333.88)	2.00 (1.56 to 2.45)
Bahrain	1203.52 (1091.35, 1332.11)	681.46 (591.17, 765.26)	-2.41 (-2.76 to -2.06)	Dominican Republic	288.02 (250.75, 333.46)	294.88 (229.69, 409.07)	0.47 (0.32 to 0.63)
Liberia	772.17 (618.68, 918.39)	652.08 (513.70, 830.56)	-0.48 (-0.65 to -0.32)	Israel	461.47 (423.92, 494.02)	292.37 (263.81, 320.70)	-1.29 (-1.45 to -1.13)
Sierra Leone	785.46 (645.47, 927.38)	646.88 (505.84, 831.93)	-0.34 (-0.49 to -0.18)	Principality of Monaco	333.93 (281.51, 385.14)	292.09 (246.10, 339.51)	-0.38 (-0.46 to -0.30)
Uruguay	641.69 (611.97, 670.73)	642.28 (600.48, 679.05)	-0.07 (-0.20 to 0.05)	Switzerland	444.04 (412.42, 477.44)	289.27 (259.16, 319.86)	-1.30 (-1.36 to -1.24)
Tajikistan	1063.76 (900.02, 1271.71)	637.12 (492.25, 826.72)	-2.04 (-2.27 to -1.82)	Republic of Korea	545.98 (472.24, 617.55)	289.13 (251.75, 335.70)	-2.09 (-2.27 to -1.90)
Senegal	784.23 (657.40, 937.47)	632.07 (513.66, 758.44)	-0.40 (-0.73 to -0.07)	Italy	444.09 (415.70, 469.61)	285.38 (256.09, 309.66)	-1.33 (-1.48 to -1.19)
Malaysia	895.86 (789.18, 1003.19)	628.09 (565.40, 692.06)	-1.67 (-1.90 to -1.44)	Chile	484.47 (462.17, 508.31)	282.02 (256.06, 301.72)	-1.29 (-1.57 to -1.01)
Kyrgyzstan	1977.09 (1839.47, 2129.62)	627.15 (542.74, 714.78)	-4.48 (-5.02 to -3.94)	Slovenia	580.81 (547.90, 617.28)	277.59 (245.36, 308.42)	-2.85 (-3.08 to -2.61)

Sudan	823.90 (425.43, 1245.79)	623.17 (442.16, 841.65)	-1.01 (-1.05 to -0.97)	Ukraine	1055.36 (999.59, 1107.94)	274.39 (225.05, 334.21)	-5.68 (-6.13 to -5.22)
Cameroon	863.65 (704.13, 1027.10)	620.09 (506.50, 770.63)	-0.94 (-1.05 to -0.83)	Slovakia	346.50 (309.90, 378.70)	272.01 (238.55, 314.65)	-0.52 (-0.63 to -0.40)
Ethiopia	1093.38 (817.17, 1295.81)	619.18 (510.02, 708.79)	-2.18 (-2.31 to -2.05)	Finland	322.94 (301.51, 347.53)	259.79 (237.73, 283.43)	-0.77 (-0.89 to -0.65)
Republic of Côte d'Ivoire	842.60 (704.68, 1004.37)	617.34 (510.65, 756.38)	-0.89 (-1.01 to -0.77)	Trinidad and Tobago	295.55 (277.46, 314.49)	256.19 (210.35, 308.60)	-0.62 (-0.76 to -0.47)
Gabon	970.41 (788.18, 1196.51)	616.69 (481.40, 822.05)	-1.54 (-1.62 to -1.47)	Lithuania	644.22 (605.75, 684.04)	249.92 (222.79, 275.46)	-3.26 (-3.42 to -3.09)
Comoros	943.55 (681.95, 1232.71)	615.46 (455.41, 774.81)	-1.59 (-1.80 to -1.37)	Belarus	1022.26 (963.46, 1084.37)	245.19 (212.95, 282.99)	-5.74 (-6.26 to -5.21)
Equatorial Guinea	1292.92 (929.68, 1703.85)	613.98 (458.71, 833.30)	-2.72 (-3.01 to -2.43)	Malta	450.93 (419.92, 483.79)	244.56 (218.62, 274.13)	-2.14 (-2.31 to -1.96)
Syrian Arab Republic	605.31 (469.60, 746.18)	605.91 (498.55, 740.44)	-0.21 (-0.31 to -0.10)	Saint Vincent and the Grenadines	150.03 (137.04, 163.29)	240.14 (216.88, 267.42)	1.24 (0.95 to 1.53)
Benin	841.84 (663.23, 993.94)	604.11 (487.65, 760.40)	-0.89 (-1.09 to -0.69)	Commonwealth of the Bahamas	210.29 (191.81, 229.66)	239.25 (201.04, 280.35)	0.25 (0.14 to 0.36)
Egypt	1015.46 (857.09, 1138.64)	602.17 (511.84, 700.27)	-1.92 (-2.12 to -1.71)	Uzbekistan	583.73 (513.50, 662.35)	238.39 (206.48, 271.83)	-3.86 (-4.5 to -3.21)
Brunei Darussalam	1140.97 (985.18, 1313.30)	595.82 (519.54, 686.73)	-1.85 (-2.02 to -1.69)	Latvia	316.05 (287.48, 345.60)	214.69 (185.89, 243.60)	-1.24 (-1.54 to -0.93)
United Arab Emirates	897.36 (701.58, 1161.39)	594.58 (488.75, 713.85)	-0.24 (-0.68 to 0.21)	France	344.81 (319.26, 368.38)	208.64 (188.85, 228.62)	-1.87 (-2.21 to -1.53)
United Kingdom of Great Britain and Northern Ireland	728.64 (699.01, 754.24)	571.45 (532.64, 601.96)	-0.65 (-0.73 to -0.57)	Peru	271.00 (214.59, 328.03)	200.04 (154.29, 255.74)	-0.86 (-1.00 to -0.72)
Colombia	763.19 (724.42, 796.84)	559.45 (477.79, 640.18)	-1.52 (-1.71 to -1.32)	Bermuda	251.73 (230.95, 273.39)	198.74 (171.34, 230.34)	-0.95 (-1.11 to -0.79)
Brazil	895.31 (848.17, 935.19)	558.69 (517.05, 588.99)	-1.96 (-2.20 to -1.72)	Montenegro	185.35 (161.06, 212.82)	187.53 (161.34, 217.71)	0.10 (0.03 to 0.18)
Morocco	505.94 (361.23, 631.38)	557.91 (461.15, 645.66)	0.45 (0.37 to 0.53)	Republic of San Marino	297.48 (259.03, 337.09)	183.81 (148.11, 222.92)	-1.00 (-1.20 to -0.80)
United Republic of Tanzania	683.23 (554.36, 796.05)	543.49 (445.23, 659.63)	-0.95 (-1.01 to -0.88)	United States Virgin Islands	266.67 (230.13, 308.34)	182.91 (149.95, 224.07)	-1.14 (-1.30 to -0.99)
Seychelles	772.86 (673.87, 857.06)	538.59 (475.57, 600.26)	-1.21 (-1.32 to -1.09)	Antigua and Barbuda	132.15 (121.23, 143.46)	179.35 (165.54, 194.02)	0.84 (0.70 to 0.98)
Netherlands	658.81 (616.49, 692.81)	537.79 (493.18, 576.92)	-0.72 (-0.85 to -0.60)	Barbados	153.44 (141.23, 166.25)	171.75 (142.62, 201.16)	0.25 (0.10 to 0.40)
Cook Islands	1066.62 (895.40, 1222.27)	534.64 (449.70, 630.68)	-2.44 (-2.59 to -2.30)	Estonia	251.93 (233.44, 271.78)	170.09 (151.14, 190.77)	-1.26 (-1.42 to -1.11)
Saudi Arabia	695.74 (529.30, 865.83)	533.08 (452.57, 618.80)	-0.92 (-0.97 to -0.88)	Kuwait	202.99 (182.25, 226.32)	160.46 (138.49, 185.65)	-0.77 (-0.96 to -0.57)
Mexico	739.18 (708.08, 763.46)	519.67 (469.83, 570.36)	-1.18 (-1.28 to -1.07)	Japan	274.06 (251.90, 296.49)	155.76 (137.62, 174.26)	-1.78 (-1.93 to -1.64)
Djibouti	704.20 (516.70, 903.30)	506.44 (362.23, 680.90)	-1.21 (-1.35 to -1.07)	Singapore	770.14 (729.76, 806.94)	146.48 (131.42, 161.67)	-5.47 (-5.59 to -5.36)

Notes: ASDR – age-standardised DALYs rate (per 100 000 population); DALYs – disability-adjusted life years; COPD – chronic obstructive pulmonary disease; EAPC – estimated annual percentage change; UI – uncertainty interval; CI – confidence interval.

Table S3. QCI of COPD at the national level in 1990 and 2021 and the EAPC from 1990 to 2021

Countries /Territories	1990	2021	EPAC (95%CI)	Countries /Territories	1990	2021	EPAC (95%CI)	Countries /Territories	1990	2021	EPAC (95%CI)
Papua New Guinea	7.45	19.18	2.74 (2.30 to 3.18)	Comoros	62.53	78.02	0.78 (0.69 to 0.87)	Iran (Islamic Republic of)	82.47	87.52	0.12 (0.09 to 0.15)
Nepal	24.09	37.41	1.17 (0.74 to 1.60)	Maldives	46.66	78.34	1.85 (1.71 to 2.00)	Bahamas	88.08	87.61	0.03 (0.00 to 0.05)
Nauru	28.97	45.43	1.79 (1.48 to 2.11)	Mozambique	75.22	78.56	-0.02 (-0.09 to 0.05)	United Kingdom	83.29	87.68	0.16 (0.14 to 0.17)
Vanuatu	34.05	46.35	1.27 (1.16 to 1.39)	South Africa	77.26	79.01	0.14 (0.04 to 0.24)	Dominican Republic	84.95	87.74	0.01 (-0.02 to 0.05)
Myanmar	31.13	47.01	1.69 (1.55 to 1.83)	Equatorial Guinea	56.64	79.27	1.24 (1.12 to 1.36)	Spain	79.91	87.75	0.32 (0.29 to 0.36)
Democratic People's Republic of Korea	36.11	47.16	0.87 (0.80 to 0.93)	Philippines	74.01	79.34	0.17 (0.15 to 0.19)	United Arab Emirates	75.94	88.03	0.12 (-0.03 to 0.27)
Marshall Islands	33.08	47.28	1.07 (0.97 to 1.17)	Tajikistan	68.48	79.60	0.66 (0.57 to 0.76)	North Macedonia	83.81	88.22	0.23 (0.20 to 0.25)
Kiribati	34.73	47.34	1.28 (1.18 to 1.39)	Australia	76.49	79.67	0.15 (0.06 to 0.24)	Azerbaijan	78.62	88.30	0.49 (0.41 to 0.57)
Palau	45.71	51.26	0.22 (0.13 to 0.30)	Denmark	80.70	79.82	0.03 (-0.04 to 0.09)	Japan	87.56	88.47	0.06 (0.03 to 0.09)
India	44.35	53.46	0.52 (0.37 to 0.67)	Brazil	65.89	79.92	0.81 (0.71 to 0.91)	Armenia	68.26	88.51	0.92 (0.81 to 1.02)
Micronesia (Federated States of)	31.59	54.64	1.89 (1.73 to 2.06)	Liberia	68.12	80.07	0.46 (0.37 to 0.54)	Portugal	84.93	88.53	0.15 (0.12 to 0.18)
Samoa	43.30	56.88	0.90 (0.79 to 1.02)	Bahrain	62.64	80.18	1.02 (0.90 to 1.14)	United States of America	91.59	88.59	-0.09 (-0.12 to -0.07)
Bhutan	48.50	58.31	0.68 (0.60 to 0.76)	Gambia	74.20	80.31	0.20 (0.13 to 0.27)	Romania	66.15	88.66	0.83 (0.71 to 0.96)
Solomon Islands	49.91	58.61	0.56 (0.52 to 0.61)	Saint Kitts and Nevis	78.80	80.34	0.04 (-0.01 to 0.08)	Trinidad and Tobago	83.29	88.77	0.23 (0.20 to 0.27)
Lesotho	62.52	59.27	-0.61(-0.90 to -0.31)	Belize	86.30	80.59	-0.11 (-0.29 to 0.07)	Italy	83.81	88.81	0.15 (0.12 to 0.18)
Uruguay	61.79	60.41	0.01 (-0.06 to 0.08)	Cook Islands	63.32	80.71	0.83 (0.77 to 0.89)	Libya	88.31	88.95	-0.05 (-0.08 to -0.02)
Tuvalu	38.16	60.95	1.55 (1.44 to 1.66)	Zimbabwe	81.20	80.73	-0.11 (-0.22 to 0.00)	Serbia	81.00	89.15	0.35 (0.32 to 0.38)
Pakistan	55.97	61.18	0.52 (0.38 to 0.66)	Chad	76.86	80.83	0.12 (0.10 to 0.14)	Croatia	89.78	89.20	-0.04 (-0.06 to -0.03)
Honduras	63.20	61.25	-0.28 (-0.39 to -0.17)	Venezuela (Bolivarian Republic of)	80.43	80.89	0.06 (0.00 to 0.13)	Burkina Faso	83.89	89.40	0.19 (0.18 to 0.21)
China	0.00	61.99	16.10 (9.59 to 22.98)	Kyrgyzstan	43.22	81.42	2.95 (2.56 to 3.33)	Germany	88.82	89.44	-0.02 (-0.06 to 0.02)
Eswatini	52.63	62.25	0.19 (-0.35 to 0.74)	Seychelles	69.29	81.71	0.52 (0.49 to 0.55)	Peru	85.76	89.64	0.15 (0.12 to 0.19)
Madagascar	51.30	62.38	0.67 (0.64 to 0.71)	El Salvador	75.66	81.88	0.24 (0.20 to 0.29)	Luxembourg	85.71	89.77	0.15 (0.12 to 0.18)
Central African Republic	51.17	62.73	0.71 (0.68 to 0.75)	Ecuador	73.54	81.93	0.16 (0.08 to 0.24)	Nigeria	83.98	90.01	0.23 (0.22 to 0.25)
Sao Tome and Principe	52.88	62.86	0.50 (0.41 to 0.58)	Yemen	77.95	81.94	0.16 (0.14 to 0.18)	Palestine	82.87	90.44	0.33 (0.29 to 0.37)
Niue	51.24	63.84	0.87 (0.81 to 0.94)	Costa Rica	76.32	81.95	0.34 (0.21 to 0.46)	Algeria	86.57	90.52	0.10 (0.07 to 0.12)

Tonga	58.84	63.92	0.21 (0.14 to 0.28)	Nicaragua	82.44	82.06	-0.11 (-0.19 to -0.04)	Andorra	83.39	90.54	0.25 (0.20 to 0.30)
American Samoa	50.77	64.05	0.84 (0.78 to 0.91)	Djibouti	69.08	82.11	0.59 (0.58 to 0.61)	Oman	88.17	90.55	0.01 (-0.02 to 0.04)
Northern Mariana Islands	52.93	64.56	0.56 (0.50 to 0.63)	Togo	73.94	82.23	0.28 (0.24 to 0.32)	Bosnia and Herzegovina	81.56	90.75	0.44 (0.38 to 0.51)
Brunei Darussalam	55.73	65.38	0.28 (0.17 to 0.39)	Guinea	74.28	82.25	0.22 (0.18 to 0.27)	Antigua and Barbuda	92.76	90.79	-0.05 (-0.07 to -0.03)
Tokelau	53.07	65.39	0.77 (0.73 to 0.81)	Guyana	84.46	82.33	-0.1 (-0.15 to -0.05)	Albania	74.51	90.86	0.58 (0.48 to 0.69)
Haiti	59.19	65.89	0.21 (0.17 to 0.26)	Cameroon	68.72	82.43	0.54 (0.50 to 0.58)	Czechia	88.67	90.98	-0.13 (-0.21 to -0.05)
Lao People's Democratic Republic	48.33	66.40	1.19 (1.13 to 1.25)	Cuba	86.74	82.56	-0.16 (-0.20 to -0.13)	Ghana	88.87	91.11	0.03 (-0.01 to 0.07)
Fiji	57.36	66.81	0.84 (0.66 to 1.01)	Guatemala	74.14	82.67	0.36 (0.26 to 0.46)	Lebanon	79.98	91.35	0.39 (0.34 to 0.43)
Namibia	56.51	66.85	0.69 (0.50 to 0.88)	Ethiopia	65.98	82.71	0.85 (0.80 to 0.91)	Bermuda	86.42	91.40	0.18 (0.15 to 0.21)
Indonesia	60.88	67.19	0.28 (0.23 to 0.33)	Taiwan (Province of China)	78.41	82.72	0.23 (0.20 to 0.27)	Barbados	91.65	91.48	0.01 (-0.01 to 0.02)
Timor-Leste	62.32	67.60	0.15 (0.08 to 0.21)	Senegal	73.33	82.72	0.3 (0.20 to 0.39)	Bulgaria	81.76	91.56	0.37 (0.34 to 0.41)
Democratic Republic of the Congo	59.60	67.93	0.43 (0.40 to 0.46)	Türkiye	71.76	82.74	0.32 (0.20 to 0.45)	Turkmenistan	70.49	91.67	1.01 (0.82 to 1.19)
Sri Lanka	44.84	68.84	1.06 (0.91 to 1.21)	Netherlands	82.66	82.82	0.11 (0.07 to 0.16)	United States Virgin Islands	83.41	91.74	0.29 (0.27 to 0.31)
Eritrea	53.55	69.14	0.81 (0.77 to 0.86)	Cabo Verde	56.68	83.02	0.96 (0.66 to 1.27)	Qatar	83.02	92.20	0.38 (0.30 to 0.46)
Kazakhstan	68.26	69.58	0.37 (0.11 to 0.63)	Paraguay	85.16	83.27	-0.14 (-0.17 to -0.12)	Republic of Moldova	68.18	92.47	1.20 (1.10 to 1.30)
South Sudan	56.91	70.00	0.72 (0.62 to 0.83)	Grenada	85.51	83.66	0.00 (-0.12 to 0.12)	Republic of Korea	80.07	92.66	0.49 (0.44 to 0.55)
Afghanistan	60.29	70.57	0.59 (0.47 to 0.71)	Panama	80.39	83.80	0.20 (0.14 to 0.25)	Slovenia	81.79	92.78	0.49 (0.43 to 0.54)
Congo	50.19	70.92	1.31 (1.22 to 1.40)	Suriname	77.08	84.00	0.24 (0.18 to 0.29)	Greece	93.58	92.92	-0.08 (-0.15 to -0.01)
Argentina	68.06	70.93	0.15 (0.04 to 0.27)	Hungary	80.01	84.05	0.08 (-0.02 to 0.19)	Austria	93.52	93.17	-0.04 (-0.07 to -0.02)
Kenya	71.05	71.11	-0.15 (-0.23 to -0.06)	Republic of Côte d'Ivoire	70.99	84.12	0.55 (0.51 to 0.58)	Iceland	93.46	93.25	-0.03 (-0.05 to -0.02)
Bangladesh	48.25	71.24	1.39 (1.28 to 1.50)	Puerto Rico	75.61	84.15	0.58 (0.48 to 0.68)	Georgia	95.18	93.35	-0.24 (-0.29 to -0.19)
Viet Nam	59.65	71.61	0.52 (0.47 to 0.57)	Saudi Arabia	67.28	84.20	0.81 (0.77 to 0.85)	Israel	88.34	93.43	0.15 (0.12 to 0.18)
Cambodia	66.17	71.99	0.29 (0.27 to 0.30)	Niger	72.59	84.25	0.41 (0.35 to 0.47)	Uzbekistan	82.09	93.61	0.62 (0.49 to 0.74)
Mali	63.38	73.01	0.32 (0.27 to 0.38)	Ireland	69.59	84.48	0.69 (0.60 to 0.78)	Monaco	93.51	93.64	-0.01 (-0.01 to 0.00)
Greenland	51.64	73.36	1.12 (1.00 to 1.23)	Thailand	63.29	84.55	1.24 (1.16 to 1.31)	Poland	85.26	93.67	0.25 (0.21 to 0.30)
Burundi	53.40	73.85	1.22 (1.11 to 1.33)	Sierra Leone	75.14	84.57	0.28 (0.24 to 0.33)	Finland	92.52	93.75	0.02 (0.00 to 0.04)
Somalia	56.35	73.95	0.85 (0.82 to 0.89)	Cyprus	68.50	84.66	0.71 (0.62 to 0.80)	Malta	87.31	93.79	0.23 (0.21 to 0.25)

Chile	67.72	74.62	0.11 (0.00 to 0.23)	Benin	72.72	84.71	0.43 (0.37 to 0.49)	Tunisia	91.97	94.09	0.12 (0.10 to 0.13)
Guinea-Bissau	59.26	74.95	0.59 (0.49 to 0.68)	United Republic of Tanzania	74.45	84.92	0.47 (0.46 to 0.49)	Russian Federation	80.05	94.14	0.72 (0.62 to 0.82)
Angola	60.33	75.75	0.85 (0.81 to 0.88)	Mongolia	68.51	85.06	1.01 (0.90 to 1.13)	Iraq	94.63	94.37	0.04 (0.02 to 0.06)
Zambia	67.41	75.81	0.48 (0.40 to 0.57)	Jamaica	88.33	85.10	-0.07 (-0.19 to 0.05)	Lithuania	78.86	94.43	0.61 (0.57 to 0.66)
Bolivia (Plurinational State of)	67.46	75.88	0.32 (0.30 to 0.34)	Singapore	57.11	85.19	1.31 (1.18 to 1.45)	Slovakia	92.65	94.60	0.03 (0.01 to 0.05)
Gabon	55.89	76.11	1.05 (1.02 to 1.08)	Sudan	78.54	85.25	0.27 (0.26 to 0.28)	Jordan	87.27	94.65	0.34 (0.31 to 0.37)
New Zealand	70.10	76.52	0.36 (0.31 to 0.42)	Norway	93.39	85.71	-0.30 (-0.34 to -0.26)	Ukraine	68.16	94.66	1.59 (1.42 to 1.77)
Malaysia	62.79	76.71	0.89 (0.77 to 1.00)	Belgium	77.57	85.76	0.38 (0.35 to 0.40)	Switzerland	91.48	94.85	0.08 (0.05 to 0.11)
Rwanda	48.79	76.72	2.07 (1.81 to 2.33)	Egypt	64.28	85.78	0.94 (0.84 to 1.04)	Sweden	98.55	94.92	-0.14 (-0.16 to -0.13)
Malawi	66.82	76.82	0.58 (0.49 to 0.66)	Guam	65.66	86.01	0.79 (0.68 to 0.90)	France	86.68	94.92	0.36 (0.29 to 0.43)
Saint Lucia	71.91	76.97	0.40 (0.31 to 0.49)	Syrian Arab Republic	84.22	86.04	0.12 (0.09 to 0.15)	Estonia	90.92	95.04	0.18 (0.16 to 0.21)
Botswana	56.69	77.01	1.11 (1.02 to 1.19)	Canada	82.14	86.25	0.11 (0.06 to 0.17)	Belarus	64.98	95.97	1.61 (1.47 to 1.76)
Colombia	70.81	77.38	0.36 (0.32 to 0.39)	Saint Vincent and the Grenadines	92.41	86.36	-0.17 (-0.22 to -0.11)	Latvia	93.20	96.65	0.10 (0.05 to 0.14)
Mexico	65.22	77.80	0.57 (0.53 to 0.62)	Mauritius	72.41	86.88	0.54 (0.48 to 0.60)	San Marino	93.92	96.93	0.04 (0.02 to 0.06)
Uganda	64.12	77.85	0.84 (0.76 to 0.91)	Morocco	84.61	87.06	0.05 (0.02 to 0.08)	Montenegro	100.00	98.06	-0.06 (-0.07 to -0.05)
Dominica	77.76	78.00	0.01 (-0.01 to 0.02)	Mauritania	71.84	87.14	0.53 (0.42 to 0.63)	Kuwait	99.24	99.50	0.00 (-0.01 to 0.02)

Notes: QCI – quality of care index; EAPC – estimated annual percentage change; COPD – chronic obstructive pulmonary disease; CI – confidence interval.

Table S4. The cross-country inequalities in ASDR of COPD in 1990 and 2021, by sex.

Sex	Health inequality metrics	year	value	95%CI
Both	Slope index of inequality	1990	-612.44	-793.80 to -431.08
		2021	-555.90	-670.85 to -440.95
	Concentration index	1990	-0.14	-0.20 to -0.09
		2021	-0.13	-0.17 to -0.08
Female	Slope index of inequality	1990	-549.63	-686.94 to -412.33
		2021	-463.17	-564.73 to -361.62
	Concentration index	1990	-0.16	-0.22 to -0.10
		2021	-0.14	-0.19 to -0.09
Male	Slope index of inequality	1990	-608.75	-863.36 to -354.13
		2021	-645.40	-786.86 to -503.94
	Concentration index	1990	-0.11	-0.16 to -0.06
		2021	-0.11	-0.15 to -0.06

Notes: ASDR – age-standardised DALYs rate (per 100 000 population); DALYs – disability-adjusted life years; COPD – chronic obstructive pulmonary disease; CI – confidence interval.

Table S5. The cross-country inequalities in QCI of COPD in 1990 and 2021, by sex.

Sex	Health inequality metrics	year	value	95%CI
Both	Slope index of inequality	1990	21.78	15.89 to 27.68
		2021	16.72	12.69 to 20.75
	Concentration index	1990	0.11	0.06 to 0.15
		2021	0.04	0.02 to 0.05
Female	Slope index of inequality	1990	21.43	16.19 to 26.67
		2021	15.34	11.52 to 19.15
	Concentration index	1990	0.09	0.05 to 0.13
		2021	0.04	0.02 to 0.05
Male	Slope index of inequality	1990	17.88	11.78 to 23.97
		2021	15.79	11.94 to 19.63
	Concentration index	1990	0.09	0.04 to 0.13
		2021	0.03	0.01 to 0.05

Notes: QCI – quality of care index; COPD – chronic obstructive pulmonary disease; CI – confidence interval.

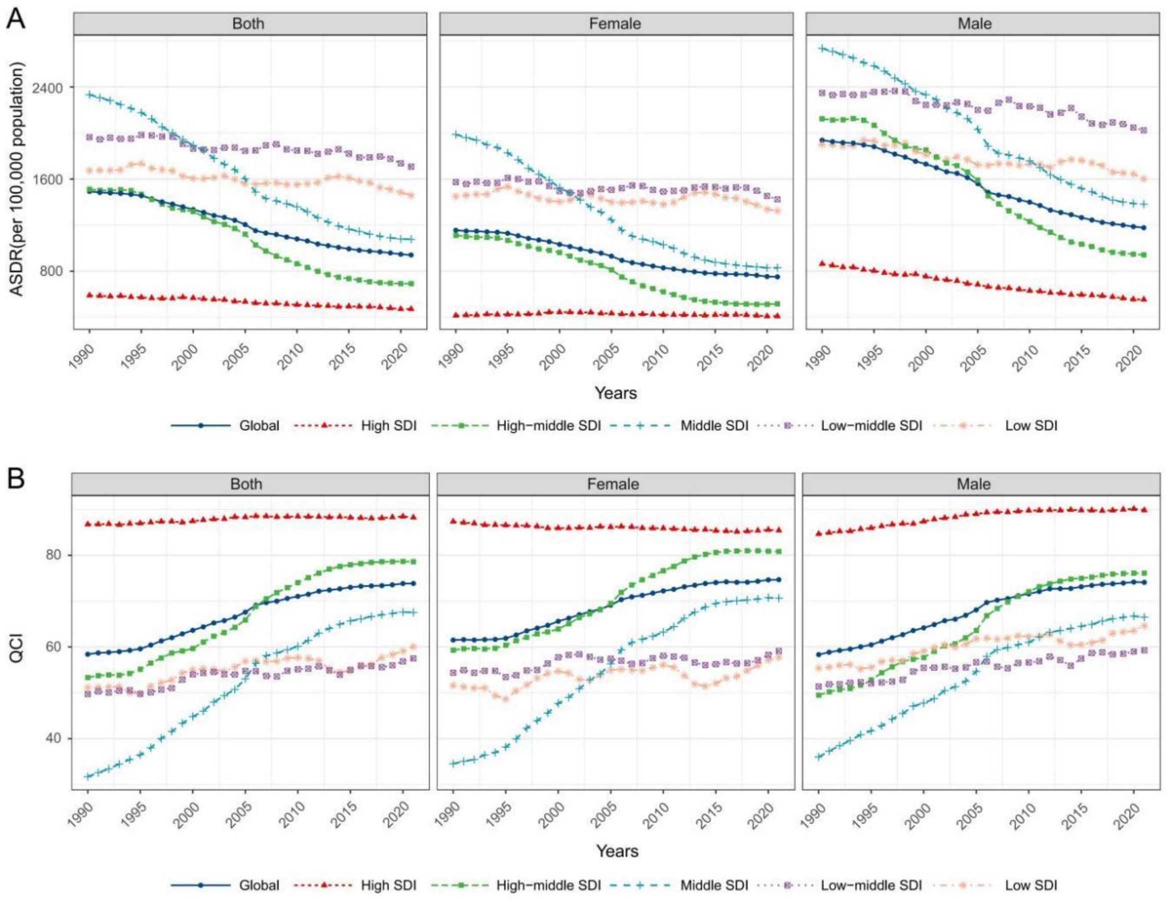

Fig S1. Year trend of ASDR (A) and QCI (B) of COPD in global and different SDI regions

Notes: ASDR – age-standardised DALYs rate (per 100 000 population); DALYs – disability-adjusted life years; QCI – quality of care index; COPD – chronic obstructive pulmonary disease; SDI – sociodemographic index.

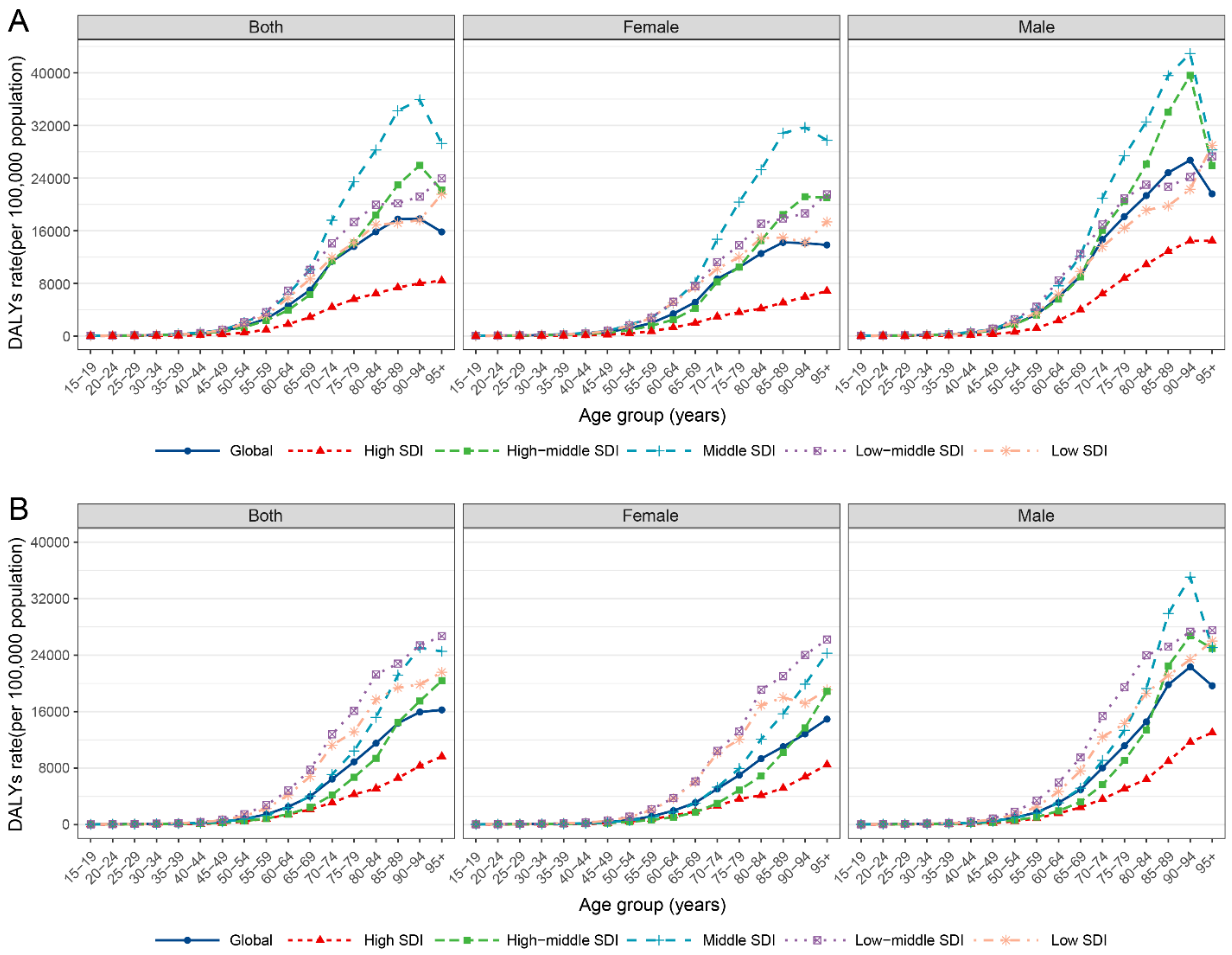

Fig S2. The age-specific DALYs rates of COPD in global and different SDI regions in 1990 (A) and 2021 (B)

Notes: DALYs – disability-adjusted life years; COPD – chronic obstructive pulmonary disease; SDI – sociodemographic index.

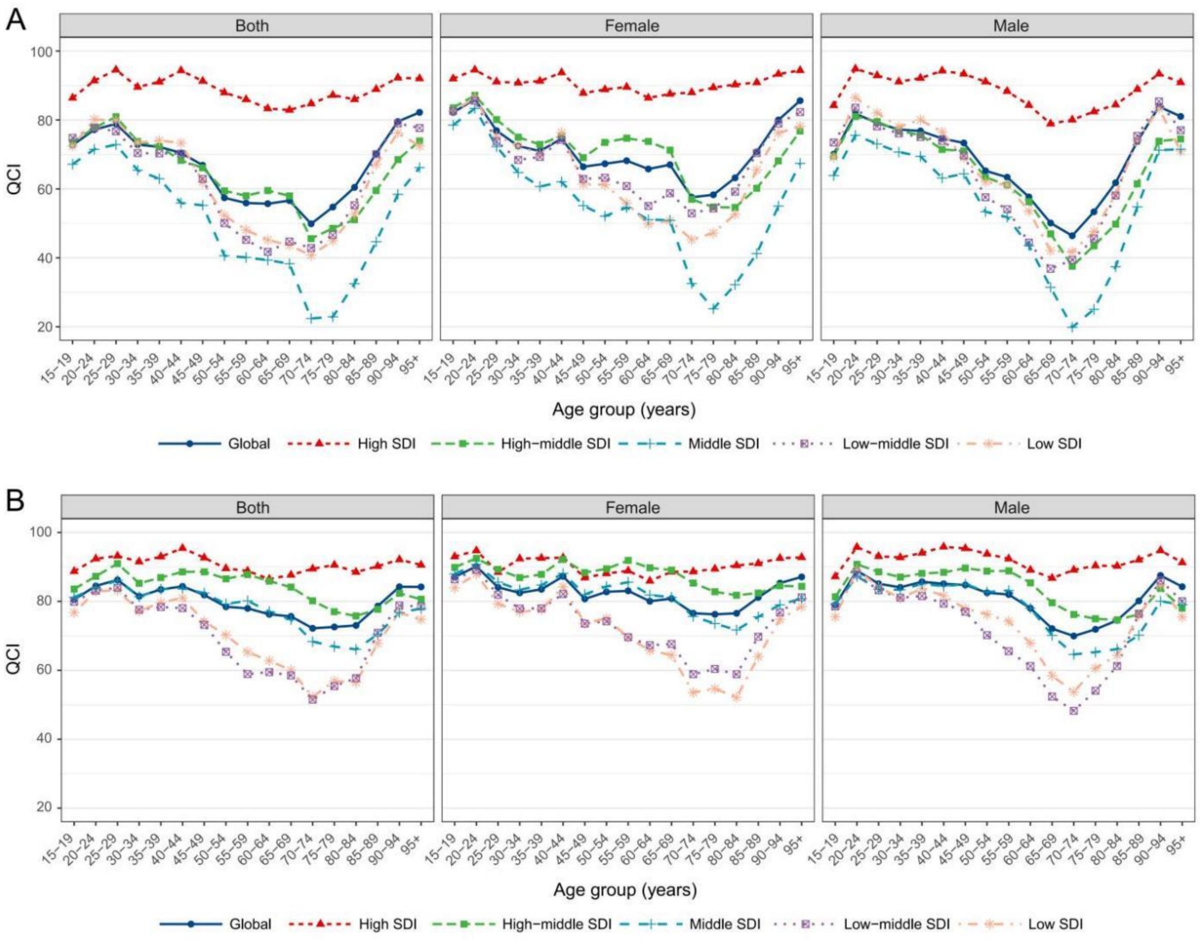

Fig S3. The age-specific QCI of COPD in global and different SDI regions in 1990 (A) and 2021 (B)

Notes: QCI – quality of care index; COPD – chronic obstructive pulmonary disease; SDI – sociodemographic index.

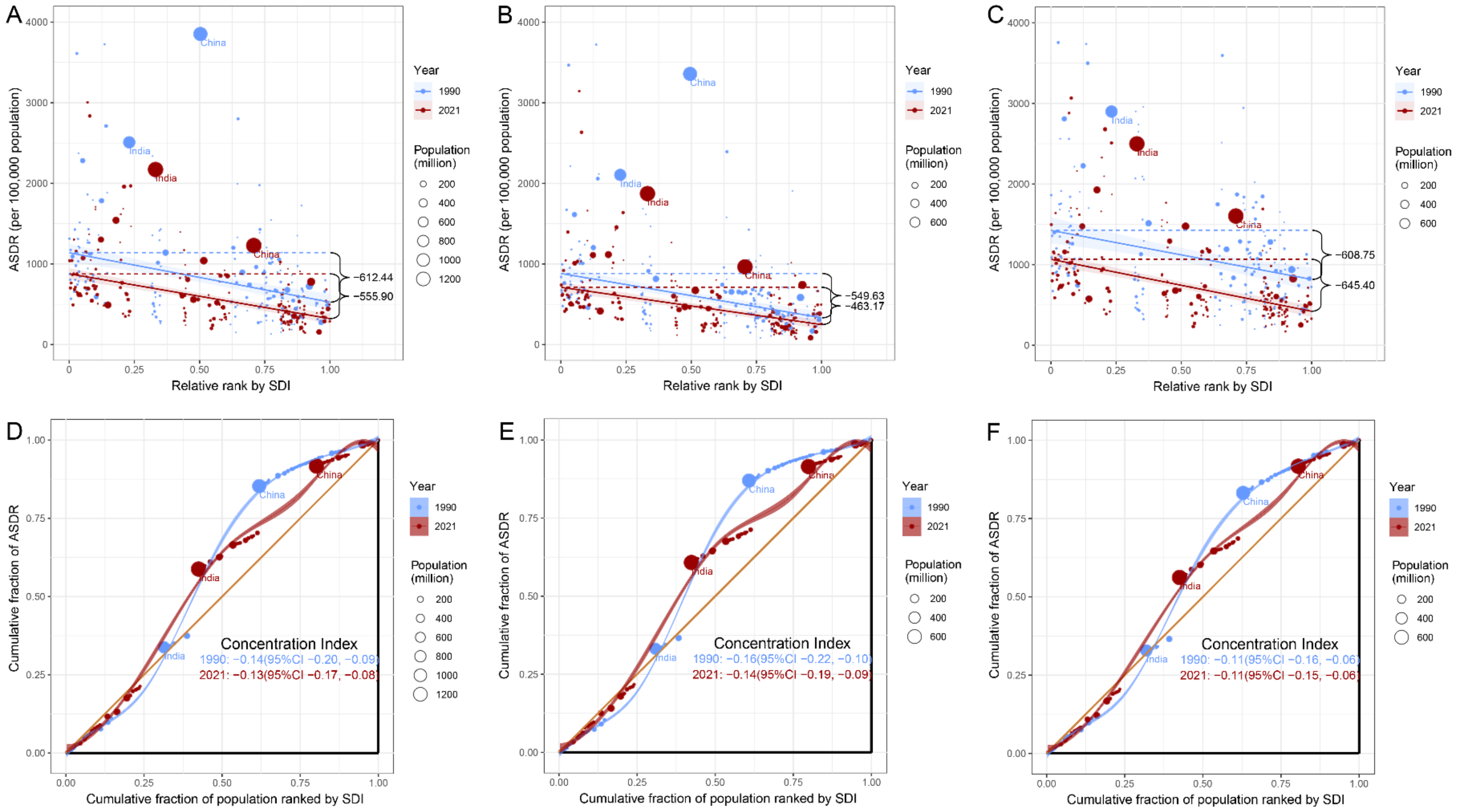

Fig S4. The cross-country inequalities in ASDR of COPD in 1990 and 2021, by sex

Notes: SII – slope index of inequality; COPD – chronic obstructive pulmonary disease; ASDR – age-standardised DALYs rate (per 100 000 population); DALYs – disability-adjusted life years; SDI, sociodemographic index. Panel A-C: SII (A. Both; B. Female; C. Male); Panel D-F: concentration index (D. Both; E. Female; F. Male).

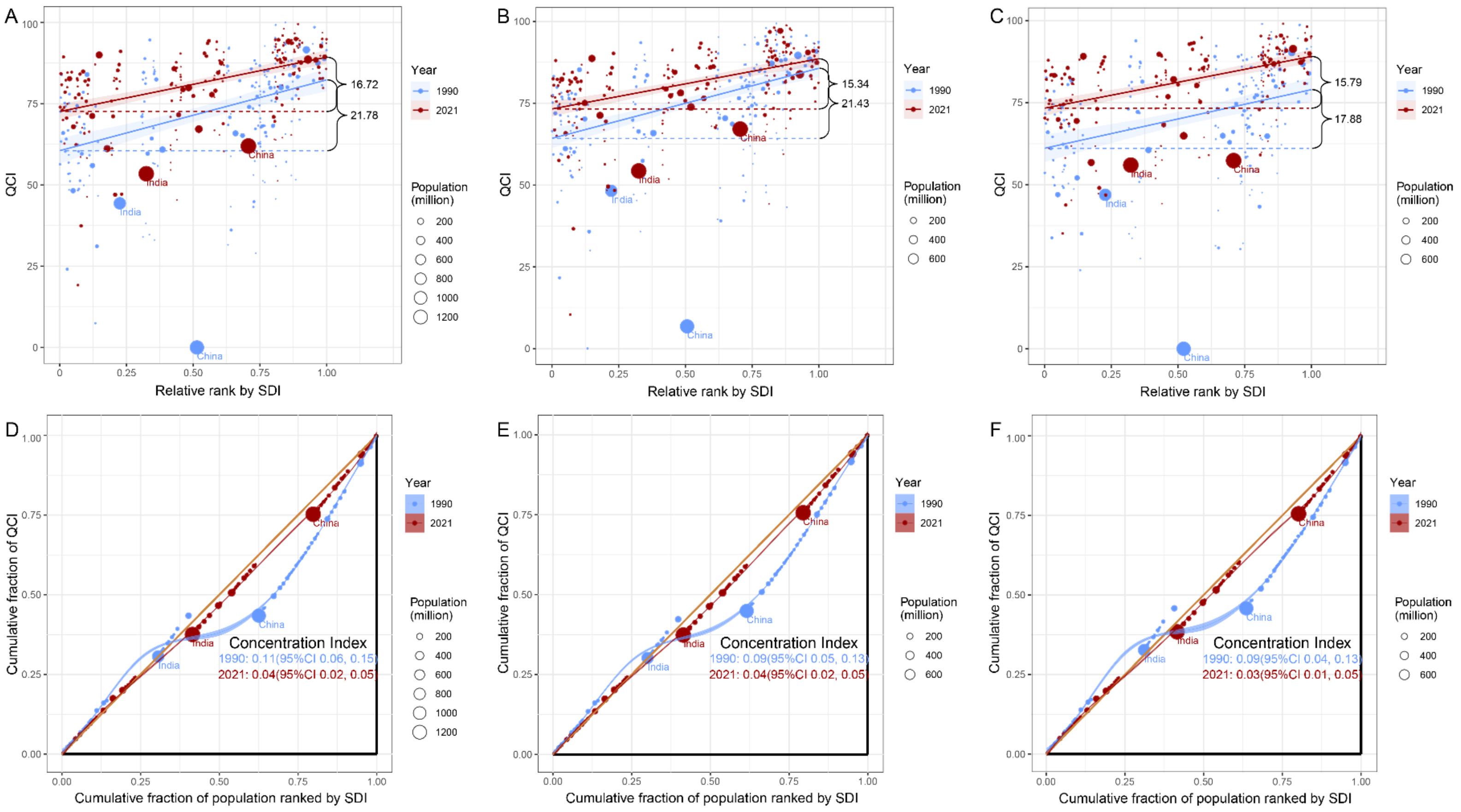

Fig S5. The cross-country inequalities in QCI of COPD in 1990 and 2021, by sex

Notes: QCI, quality of care index; SII, slope index of inequality; COPD, chronic obstructive pulmonary disease; SDI, sociodemographic index. Panel A-C: SII (A. Both; B. Female; C. Male); Panel D-F: concentration index (D. Both; E. Female; F. Male).
